# Point-of-care lung ultrasound in COVID-19 patients: inter- and intra-observer agreement in a prospective observational study

**DOI:** 10.1038/s41598-021-90153-2

**Published:** 2021-05-21

**Authors:** Markus H. Lerchbaumer, Jonathan H. Lauryn, Ulrike Bachmann, Philipp Enghard, Thomas Fischer, Jana Grune, Niklas Hegemann, Dmytro Khadzhynov, Jan Matthias Kruse, Lukas J. Lehner, Tobias Lindner, Timur Oezkan, Daniel Zickler, Wolfgang M. Kuebler, Bernd Hamm, Kai-Uwe Eckardt, Frédéric Muench

**Affiliations:** 1 Department of Radiology, Charité - Universitätsmedizin Berlin, Corporate Member of Freie Universität Berlin, Humboldt-Universität Zu Berlin, Berlin, Germany; 2 Institute of Physiology, Charité - Universitätsmedizin Berlin, corporate member of Freie Universität Berlin, Humboldt-Universität Zu Berlin, Berlin, Germany; 3 German Center for Cardiovascular Research (DZHK), Partner Site, Berlin, Germany; 4 Department of Emergency Medicine (CVK, CCM), Charité - Universitätsmedizin, Berlin, Germany; 5 Department of Nephrology and Medical Intensive Care, Charité - Universitätsmedizin Berlin, Charitéplatz 1, 10117 Berlin, Germany; 6 Center for Systems Biology, Massachusetts General Hospital Research Institute, Harvard Medical School, Boston, USA; 7 The Keenan Research Centre for Biomedical Science at St. Michael´S, Toronto, Canada; 8 Departments of Surgery and Physiology, University of Toronto, Toronto, Canada

**Keywords:** Respiratory signs and symptoms, Respiratory distress syndrome, Viral infection

## Abstract

With an urgent need for bedside imaging of coronavirus disease 2019 (COVID-19), this study’s main goal was to assess inter- and intraobserver agreement in lung ultrasound (LUS) of COVID-19 patients. In this single-center study we prospectively acquired and evaluated 100 recorded ten-second cine-loops in confirmed COVID-19 intensive care unit (ICU) patients. All loops were rated by ten observers with different subspeciality backgrounds for four times by each observer (400 loops overall) in a random sequence using a web-based rating tool. We analyzed inter- and intraobserver variability for specific pathologies and a semiquantitative LUS score. Interobserver agreement for both, identification of specific pathologies and assignment of LUS scores was fair to moderate (e.g., LUS score 1 Fleiss’ κ = 0.27; subpleural consolidations Fleiss’ κ = 0.59). Intraobserver agreement was mostly moderate to substantial with generally higher agreement for more distinct findings (e.g., lowest LUS score 0 vs. highest LUS score 3 (median Fleiss’ κ = 0.71 vs. 0.79) or air bronchograms (median Fleiss’ κ = 0.72)). Intraobserver consistency was relatively low for intermediate LUS scores (e.g. LUS Score 1 median Fleiss’ κ = 0.52). We therefore conclude that more distinct LUS findings (e.g., *air bronchograms*, *subpleural consolidations*) may be more suitable for disease monitoring, especially with more than one investigator and that training material used for LUS in point-of-care ultrasound (POCUS) should pay refined attention to areas such as *B-line* quantification and differentiation of intermediate LUS scores.

## Introduction

The novel severe acute respiratory syndrome coronavirus 2 (SARS-CoV-2) causing coronavirus disease 2019 (COVID-19) has led to a global pandemic^1,2^. SARS-CoV-2 initially affects the respiratory system with a very heterogeneous clinical presentation ranging from none or minimal symptoms to significant hypoxia due to viral pneumonia and development of an acute respiratory distress syndrome (ARDS).

While lung involvement in SARS-CoV-2 infection is primarily detected by non-enhanced computed tomography (CT)^3^, a bed-side imaging modality for frequent monitoring of disease progression would be desirable, in particular in settings where capacities for patient transport and CT imaging of infectious patients are limited^4,5^. This may be especially true for health systems in countries that have become severely affected by COVID-19, either due to general lack of access to health care or in health systems, which exceeded their capacities^6^. Ultrasound (US) investigations pose a great advantage due to their widespread availability and cost effectiveness potentially allowing more patients to receive access to imaging of the lung^7,8^. Additionally, lung ultrasound (LUS) has emerged over the last two decades as a non-invasive tool for the fast differential diagnosis of pulmonary diseases and is now used in different settings in intensive care^9,10^. Several general LUS protocols for standardized reporting and interpretation of abnormal lung findings or assessment of lung aeration have already been published and shown by way of example to be superior to conventional chest radiographs in the detection of community acquired pneumonia^11–14^.

Since lung involvement in COVID-19 is typically peripherally in location, LUS may be particularly suitable for lesion detection and follow-up of patients with COVID-19^15–17^. Furthermore, as a point-of-care ultrasound examination, LUS can be performed at the bedside by treating physicians, providing immediate information on the patient’s condition.

LUS is assumed to be an operator-dependent modality, as interobserver variabilities may influence detection and interpretation of specific findings in patients^18^. Furthermore, previous studies have demonstrated a high accuracy for LUS compared to conventional radiographic imaging, such as chest x-rays or CT imaging^19,20^. Therefore, we conducted a prospective observational study specifically of LUS in COVID-19 patients focusing on inter- and intraobserver agreement of the simultaneous interpretation of distinct LUS findings and with specific attention on eliminating potential biases. Additionally, we tested the influence of different background expertise on the detection and rating of abnormalities and potential learning effects over time.

## Methods

### Study cohort

The study was approved by the local ethics committee for COVID-19-related research and conformed to the amended Declaration of Helsinki (ethics approval reference number: EA2/066/20, *Charité COVID-19 Research Board* at the Institutional ethics committee Charité Universtitätsmedizin, Berlin, Germany). Written informed consent of all COVID-19 patients were acquired for all general COVID-19-related research projects (imaging, biomarkers, clinical findings etc.), which includes this study project. All decisions related to the diagnostic and therapeutic management of patients were made by the physicians involved in their treatment and not influenced by the study protocol at any time. The results of LUS analyses were not used to guide patient management.

All patients included in the analysis were admitted to an interdisciplinary ICU solely dedicated to the treatment of COVID-19 patients. The patients included in our analysis had at least two positive PCR tests for SARS-CoV-2 (nasal swabs, bronchial secretion, or bronchoalveolar lavage fluid) and underwent LUS at different stages of their disease. Exclusion criteria for standardized LUS assessment were prone positioning, chest drain (and/or pneumothorax), and non-adherence (e.g. delirious patients).

At the time of image acquisition (May 2020), 18 patients were treated for COVID-19 in aforementioned ICU of our hospital. Five of them were excluded from LUS (two due to thoracic drains, one for being delirious and actively opposing the examination, and two were in the immediate process of being transferred to a regular ward). Baseline patient characteristics are presented in the supplementary material (Table S1).

### LUS image acquisition and selection

Lungs were examined in the grayscale B-mode with a hand-held (tablet-like) POCUS system using a 1–6 MHz convex array transducer (Viamo sv7; Canon Medical Systems Corporation, Tochigi, Japan). All examinations were performed at the bedside by an experienced radiologist specialized in diagnostic and interventional US. The ultrasound preset was optimized for LUS, and no cosmetic filters such as compounding, spatial reduction, or harmonic imaging were used. All examinations were performed with a frame rate of 69 frames per seconds and a penetration depth of 13 cm to allow for identification of B-lines. The focal point was placed on the pleural line to increase resolution of pleural pathologies, and no multi-focusing setting was used.

**Figure 1:**
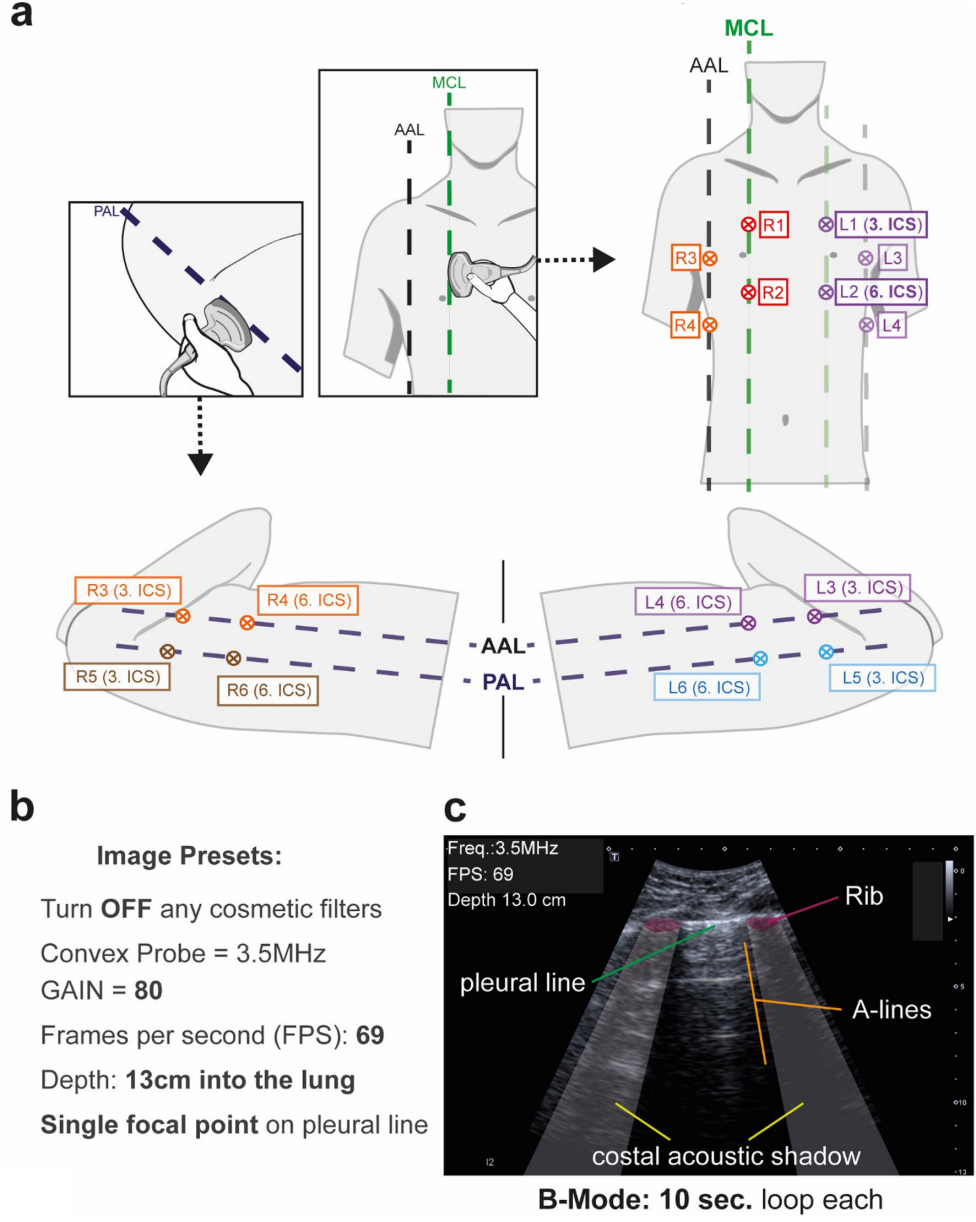
Standard operating procedure (SOP) for image acquisition. (**A**) Lung ultrasound (LUS) regions of interest for standardization of image acquisition; Points L1-L6 and R1-R6 located in the midclavicular (MCL), anterior axillary (AAL) and posterior axillary line (PAL) in the 3rd & 6th intercostal spaces (ICS) (**B**) Ultrasound imaging presets defined by SOP. Cine-loops were recorded as B-mode images for 10 s each. (**C**) Physiological LUS acoustic window confined by ribs and their corresponding shadows.

All patients were examined in supine position using a 12-point US protocol including examination of the anterolateral and posterior lung fields bilaterally. The transducer was positioned longitudinally in a 90°-degree angle to the body surface with two adjacent ribs captured in each image to allow optimal visualization of the pleural line. Three anatomic lung fields (anterior, lateral, and posterior) were identified using the midclavicular line and the anterior and posterior axillary lines as landmarks respectively. If possible, depending on patient positioning, six intercostal spaces (ICS) were examined per hemithorax. Accordingly, the transducer was placed on the 3rd and 6th ICS in the midclavicular line (R1-R2, L1-L2), on the anterior axillary line (R3-R4, L3-L4), and on the posterior axillary line (including the PLAPS [posterolateral alveolar and/or pleural syndrome] points; R5-R6, L5-L6; Fig. 1)^11^.

### Observers and image analysis

Overall, ten observers from three different medical specialties (intensive care medicine [n = 4], emergency medicine [n = 3], and physiology [n = 3]) participated in the rating of LUS images. Observers included seven physicians (six board-certified) with extensive bedside clinical experience (12 ± 6 years) and three researchers with expertise in LUS in rodent models from the Institute of Physiology^21^. Each observer had performed more than 1000 ultrasound examinations and more than 100 LUS examinations.

All LUS 10-s cine-loops were prospectively collected and stored. Overall, 144 US cine-loops from 13 patients were acquired and underwent pseudonomyzation. A radiologist, who was not involved in the study as an observer, selected 100 loops with sufficient image quality for further analyses. Selected loops were uploaded to a specifically designed online rating tool on a server accessible via personal log-in. All cine-loops were quadrupled and consecutively arranged in a random order, individually for each observer to evaluate interobserver and intraobserver agreement. As each observer would rate the identical quadrupled cine-loop 4 times, the first assessment was named *instance 1*, the second *instance 2*, the third *instance 3* and the fourth *instance 4.* Consequently, each observer would thus rate 400 cine-loops in total. Observers were allowed to view each 10-s cine-loop repeatedly for reliable rating until submitting their final decision through the online-tool. Once submitted, the loop and its rating could not be viewed again in order to prevent intraobserver bias that would result from allowing observers to reconsider previous ratings. Observers could access and leave the online-tool at any given time and pause whenever and as long as they wanted to.

**Figure 2:**
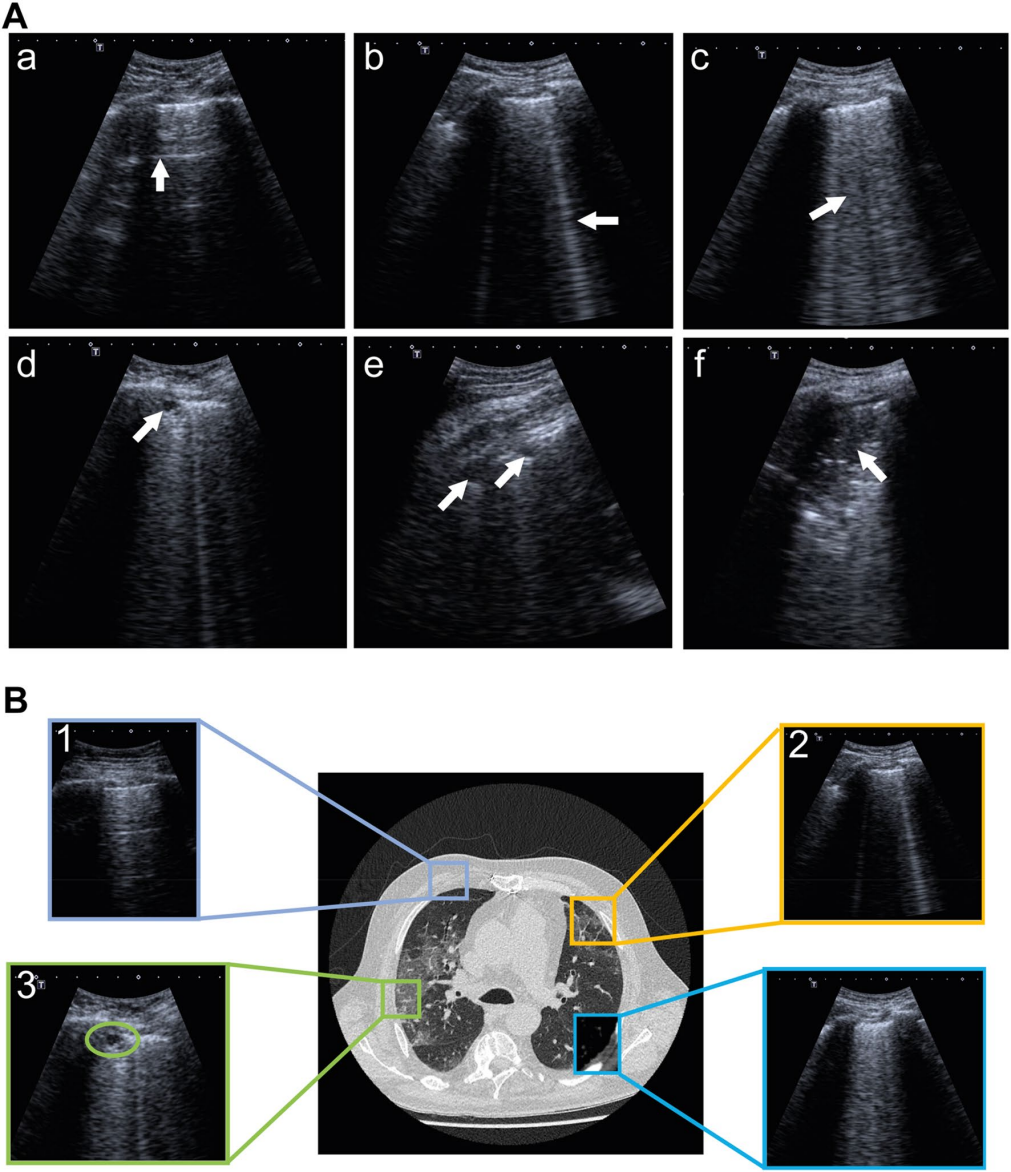
Representative images illustrating pathological LUS findings. (**A**) Typical LUS findings in COVID-19 are indicated by arrows: (**a**) A-lines; (**b**) Single B-lines; (**c**) Confluent B-lines; (**d**) Subpleural consolidations; (**e**) Substantial consolidations and pleural fragmentation; (**f**) Consolidation with air bronchogram. (**B**) Aforementioned LUS findings and their correlating computed tomography (CT) findings: (1) physiological bat sign with A-lines; (2) single B-lines; (3) Subpleural consolidation; (4) Pleural thickening/fragmentation and confluent B-lines.

The tool offered multiple-choice options with predefined answers for rating. Options included typical COVID-19-associated LUS findings (*pleural thickening/fragmentation*, *presence of B-lines* subclassified in single or < 4 B-Lines versus confluent or ≥ 4 B-Lines, *subpleural consolidations,* and *positive air bronchogram* or none of the aforementioned pathologies; Fig. 2). Air bronchograms, which are a consequence of perturbation of the air-fluid relationship in the lung parenchyma, can be visualized as arborising tubular structures representing the bronchial tree due to fluid-filled alveoli serving as acoustic medium for ultrasound waves. They are less COVID-19 specific but were included due to their common mentioning in general LUS for depicting consolidations. Of note, four patients of our cohort were simultaneously treated for a suspected bacterial pneumonia, which might show some LUS features less common in COVID-19 such as bronchograms.

Additionally, a previously described semiquantitative scoring system for assessment of lung aeration consisting of a 4-point scale based on different US patterns (Table 1) was used and graded separately from single pathologies detection by all observers^13^. The time recording for evaluation of each cine-loop was then logged by the online-tool.

**Table 1:** Lung ultrasound (LUS) score.

LUS score	Corresponding US pattern
0	normal aeration (= A-lines and up to 2 B-lines/ICS)
1	moderate loss of aeration (= multiple single B-lines/ICS)
2	severe loss of aeration (= multiple coalescent B-lines/ICS)
3	complete loss of aeration (= tissue-like pattern, consolidation, air bronchograms)

Semiquantitative LUS scoring based on four different grades with regard to aeration of the lung.

No specific training or priming was conducted prior to the rating sessions, and all observers assessed the cine-loops independently according to their own experience in clinical or pre-clinical LUS to simulate the routine clinical situation. Observers were informed that enrolled patients were admitted to the ICU with confirmed COVID-19 infection, but not about their medical condition, in particular not about their disease stages.

For additional assessment of accuracy, an agreement rating of the 100 cine loops was defined by two highly experienced radiologists in consensus (one of them European Federation of Societies for Ultrasound in Medicine and Biology level 3).

### Statistical analysis

Unless indicated otherwise, results are presented as median (IQR Q1–Q3), mean ± 95%confidence interval, or frequency (percentage of total). Statistical analysis was performed, where appropriate, using Fleiss’ kappa, Kruskal–Wallis test, and posthoc Dunn’s test with Bonferroni-adapted p-values, Pearson’s chi-square test with posthoc χ2-corrected residues and Bonferroni-corrected p-values and Cochrane’s-Q-Test and multiple McNemars tests with Bonferroni correction for determining intraobserver differences over 4 viewing instances (= #1, #2, #3, #4) and the consensus answer (= C), hypothesizing potential learning effects in single observers over time.

Fleiss’ kappa (κ) was estimated for multiple observers to determine the degree of intraobserver agreement, after correction for agreement by chance, between all four instances of the quadrupled cine-loops, as well as interobserver agreement between multiple raters, independently for each instance. Kappa values were interpreted according to Landis and Koch with κ < 0.00 corresponding to poor agreement, κ = 0.00‒0.20 to slight agreement, κ = 0.21‒0.40 to fair agreement, κ = 0.41‒0.60 to moderate agreement, κ = 0.61‒0.80 to substantial agreement, and κ = 0.81‒1.00 to almost perfect agreement^22^. A two-sided significance level of α = 0.05 was defined to indicate statistical significance.

All analyses were performed using GraphPad Prism 8 (GraphPad Software, La Jolla, CA), SPSS Statistics 26 (IBM Corp., Armonk, NY) and Excel v16.38 for MacOS (Microsoft, Redmond, WA).

In this study, we generally followed the guidelines for reporting reliability and agreement studies (GRRAS) as proposed by Kottner et al.^23^.

## Results

### Frequencies of LUS Score ratings and detected pathologies

According to radiologic consensus ratings, out of 100 cine-loops from 13 patients admitted to the ICU, 28 cine-loops were rated with LUS score 0, 20 images as LUS score 1, 38 images as LUS score 2 and 14 cine loops as LUS score 3. Fifteen cine-loops did not show any pathologies and 56 images exposed pleural thickening. Thirty-three cine-loops were characterized by single B-Lines, while 42 images exhibited confluent B-Lines (≥ 4 B-Lines). By comparison, subpleural consolidations were only seen in 22 images, while air bronchograms were even less common and only seen in 14 cine-loops.

### Interobserver and intraobserver agreement

**Figure 3:**
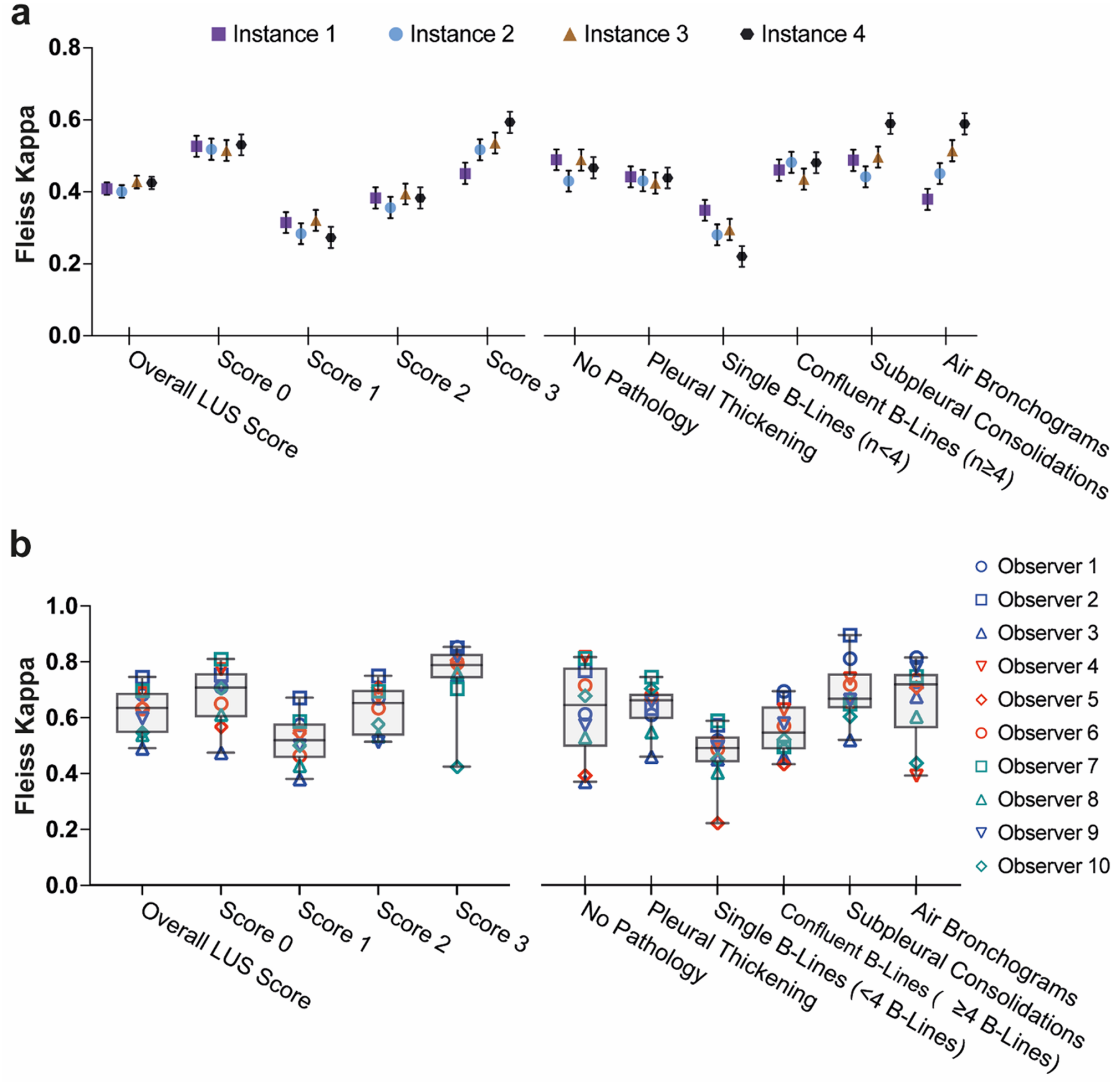
Interobserver (**a**) and intraobserver (**b**) agreement measured by Fleiss-Kappa between observers. For interobserver (**A**) last assessment of the quadrupled cine-loops (= instance 4) resulted in median κ = 0.41 (95% CI 0.39–0.43) for overall LUS score, κ = 0.53 (95% CI 0.50–0.56) for LUS score 0, κ = 0.27 (95% CI 0.24–0.30) for LUS score 1, κ = 0.38 (95% CI 0.35–0.41) for LUS score 2, κ = 0.59 (95% CI 0.56–0.62) for LUS score 3, κ = 0.47 (95% CI 0.44–0.50) for no pathology, κ = 0.44 (95% CI 0.41–0.47) for pleural thickening/fragmentation, κ = 0.22 (95% CI 0.19–0.25) for single B-lines (n < 4), κ = 0.48 (95% CI 0.45–0.51) for confluent B-lines (n ≥ 4), κ = 0.59 (95% CI 0.56–0.62) for subpleural consolidations, and κ = 0.59 (95% CI 0.56–0.62) for air bronchogram respectively. For intraobserver (**B**) over all four assessments with median κ = 0.63 (IQR 0.54–0.69) for total LUS score, median κ = 0.71 (IQR 0.6–0.76) for LUS Score 0, median κ = 0.52 (IQR 0.46–0.58) for LUS Score 1, median κ = 0.65 (IQR 0.53–0.7) for LUS Score 2 and median κ = 0.79 (IQR 0.74–0.83) for LUS Score 3. In terms of single pathologies, intraobserver agreement showed median κ-values of 0.65 (IQR 0.5–0.78) for no pathology, 0.66 (IQR 0.59–0.69) for pleural thickening; 0.49 (IQR 0.44–0.53) for single B-lines; 0.55 (IQR 0.49–0.64) for confluent B-lines; 0.67 (IQR 0.63–0.76) for pleural consolidations and 0.72 (IQR 0.56–0.76) for air bronchograms (p < 0.005 for all, cf. supplementary results for specific Fleiss Kappa values). All variabilities are color- and symbol-coded for the respective observer as well as observer group.

Interobserver agreement in LUS scores and detection of single pathologies grading resulted in Fleiss’ kappa values of fair to moderate agreement (Fig. 3A; p < 0.0005 for all; Tables S2, S3). Interobserver agreement increased with number of replications for the detection of air bronchograms and subpleural consolidations, while agreement for single B-lines decreased.

Intraobserver agreement, measured as Fleiss’ Kappa coefficient, featured largely moderate to substantial agreement among the four instances in all observers (Fig. 3B; p < 0.0005 for all; Table S4).

### Group comparison of LUS scores and single pathology detection

The time needed for evaluation per cine-loop differed significantly among observer groups, with researchers from the physiology department taking longest (reported median in sec (+ IQR) per observer group: intensive care = 19.8 (15.0–27.1); emergency medicine = 22.7 (16.7–33.3); physiology = 34.7 (21.3–70.0), Kruskal–Wallis test, p < 0.0005, Fig. S1).

**Figure 4:**
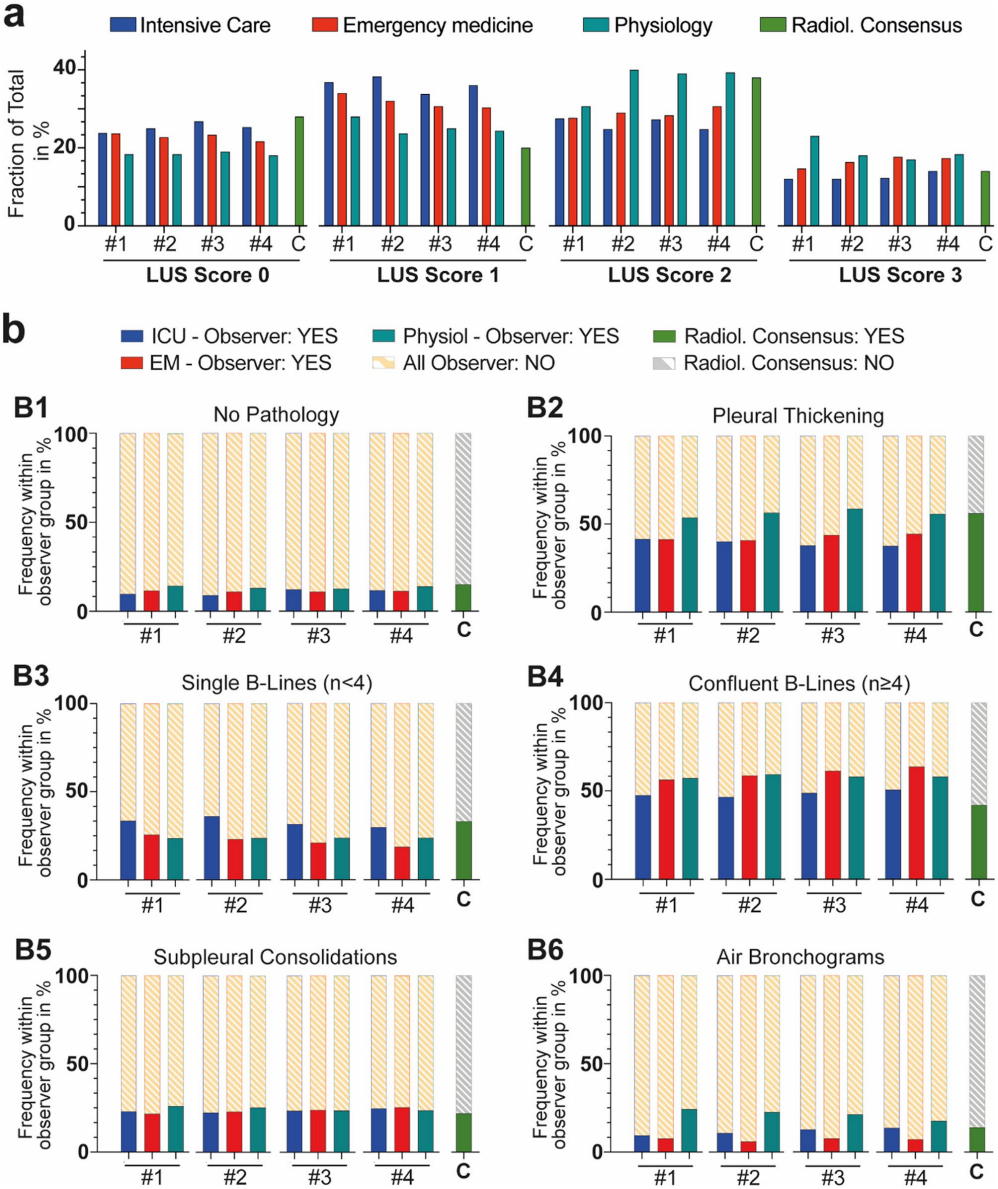
Comparison over four instances (= #1–#4) of observer groups regarding semiquantitative LUS scores (**a**) and detection of single pathologies **(b)**. (**A**) LUS score—response frequencies of each observer group as fraction of total (percentage) observers regarding their LUS scoring. Group comparison via Kruskal–Wallis test revealed a significant difference in the distribution of LUS scores in all instances between observer groups. (**B**) Detection of individual COVID-19-associated lung pathologies in LUS—Graphic representation of response frequency within observer groups as percentage over four viewing instances compared to radiologic consensus frequency (cf. supplementary results for specific statistics).

As shown in Fig. 4, the frequency of identification of individual lung abnormalities was highly constant within each of the three observer groups over all four instances. With regard to single LUS findings, ICU physicians, in accordance with radiological consensus, tended to grade B-lines less often as confluent B-lines, and observed pleural thickening less frequently compared to emergency physicians and physiology researchers (p < 0.05).

### Intraobserver learning effect over time for single pathologies

Intraobserver learning effects over four viewing instances (= #1, #2, #3, #4) and the consensus answer (= C) occurred primarily in more distinct LUS findings like subpleural consolidations and air bronchograms as seen with statistical significance especially for physiology observers improving their corresponding ratings (Figs. S31-s, 2-s, 3-s).

Further results and additional statistical analysis are presented in supplementary materials.

## Discussion

The main findings of this systematic, prospective analysis of intra- and interobserver agreement in LUS interpretation are i) fair to moderate interobserver agreement among all readers, ii) moderate to substantial intraobserver agreement, and iii) higher disagreement for some pathologies, especially pleural thickening and B-line patterns.

During COVID‐19 progression, changes in lung parenchyma are predominantly located in peripheral lung regions—identified as ground-glass opacities—in the early phases and larger subpleural consolidations or loss of aeration in basal lung regions in late disease^3,24^. In line with those peripheral changes studies have shown that highest agreement in single patient evaluation in COVID-19 using LUS can be obtained by focusing on posterior and at least ten, or better twelve, scanning positions^25,26^. Additionally, Bonadia et al. were able to demonstrate a 20% cut-off value of percental pathological area needed, in which LUS turns out to be positive in concordance with chest x-rays^27^. As in other applications of ultrasound, the depiction, quantification, and interpretation of specific findings in LUS are subjective and depend on the individual investigator^9^. Considerable experience is required for operators to generate standardized and reproducible ultrasound images or cine-loops for adequate follow-up imaging. There are two sources of variability: (I) related to generating the images and (II) related to the interpretation of those images; our study systematically studied the latter, whereas the former was minimized by having the same individual with high experience recording the images.

Our analysis shows fair to moderate interobserver agreement using a semiquantitative LUS scoring system and for detection of specific lung alterations in COVID-19. We found higher agreement among observers for the more distinct findings (e.g., LUS score 0, LUS score 3, *no pathology* and *subpleural consolidations/air bronchogram*), whereas even intraobserver consistency was fairly low for scores of 1–2 and counting of B-lines. Accordingly, interobserver agreement was lowest for *single B-lines (*< *4 B-lines)* and highest for *subpleural consolidations* and *air bronchograms*.

As compared to a radiology consensus, ICU observers tended to interpret B-lines more accurately, while physiology researchers and emergency physicians more often categorized B-lines as confluent rather than single. This tendency became even stronger over the course of viewing instances, probably explaining the poorer than expected overall inter- and intraobserver agreement (Fig. 4). We assume that ICU observers have greater clinical experience with patients with severe ARDS or cardiogenic edema and their corresponding LUS findings, especially compared to scientists whose experience relies on LUS in rodents. ICU observers, on the other hand, differed from the latter two groups regarding the identification of pleural thickening (Fig. 4). A potential reason for this might be the generally low clinical value of this specific lung finding in terms of treatment decisions, resulting in less attention to this LUS finding in clinical practice so far and generally faster grading.

Our results exposed moderate agreement of observers with different clinical backgrounds and similar accuracy compared to the consensus rating by radiological experts in US. These findings may well represent typical workflow in daily clinical routine, where LUS is performed by treating physicians from different specialities rather than specialized experts in the field of LUS, who would potentially have higher agreement due to longer experience in this special subfield of US. So far studies have shown a very heterogenous level of agreement between observers. While some studies reported almost perfect agreement between mostly two observers, other studies have revealed a rather heterogenous level of agreement. Furthermore, most mentioned studies only focused on single pathologies or single patient evaluation in general, but rarely turned the spotlight on assessing observer agreement in the simultaneous detection of multiple numbers of pathologies^19,27–29^. While Nazerian et al. nicely revealed the accuracy of LUS compared to CT images and even revealed a high interobserver variability for detecting lung consolidations, their study lacks the comparison of simultaneous detection of multiple numbers of pathologies. Also, their reported high interobserver variability might be biased, as their eight different observers were not blinded to the patients’ symptoms and condition^28^.

In a recently published study investigating interobserver agreement of LUS in COVID-19 (although without measuring intraobserver agreement), Kumar et al. found lower agreement for consolidations, similar agreement for pleural thickening and higher agreement especially when assessing B-lines^18^. This is in line with our assumption that low agreement in B-Line quantification might be due to a lack in specific training as observers met for a one hour calibration session before grading in the aforementioned study.

We conclude that—as long as observers have some experience in LUS—no specific clinical background is needed for scoring the findings, even though specific expertise is often reported as a requirement^30^. As demonstrated by Rouby and colleagues in the assessment of LUS patterns in critical ill patients, there was a sufficient learning curve for residents with little experience by the use of a short and easy-to-implement training program, supervised by a physician with expertise in bedside LUS^31^. Our data regarding learning curves supports this finding, as the least experienced researchers in regard to LUS in human patients and specific findings like air bronchograms, improved significantly, even without supervision or feedback, already after only four viewing instances.

Although the observers in our study were largely experienced in LUS in general, especially B-Line quantification may hamper the standardization of scoring without proper training and experience. Thus, standardized training programs with generally used terminology could improve the scoring of image findings. Another approach might be the usage of automated B-line quantification through deep learning algorithms^32^. Distinguished findings such as subpleural consolidations and pleural thickening resulted in a higher intra- and interobserver agreement, but a standardized approach in description and scoring of B-Lines is necessary for monitoring of patients, as these subjective findings are of greater importance for short-term follow up.

Our findings support the feasibility of LUS in general when performed as a standardized examination by physicians with different clinical backgrounds. Availability is such that it is easy to perform more LUS examinations to meet a growing demand as in the current COVID-19 pandemic^9^. Other advantages include that no staff time is needed for the transport of ICU patients to the radiology suite and that the risks associated with the transport of unstable patients are eliminated when the examination can be performed at the bedside^15^. Overall, LUS has the advantage of providing direct feedback about lung injury or disease progression to the treating physician. Finally, LUS supports other clinical parameters and can be performed more frequently than CT without concerns regarding radiation exposure.

### Strength and limitations

To our knowledge, this is one of the first large randomized analysis of inter- and intraobserver variability for LUS in general and LUS in COVID-19 in particular. The innovative web-based setup with its randomized quadrupled presentation of LUS cine-loops for each observer and the prohibition of retrospective cine-loop reviews prevented intra- and interobserver bias in this prospective observational study. Moreover, inter-and intraobserver agreement based on four instances may have had an impact on agreement, which is often based on two instances in other studies.

There was no feedback from the rating tool in regard to cases in which a LUS score would not correlate adequately with the individual pathological findings, to minimize potential observer bias through the rating tool. While this remains an important goal in a reliability study, in clinical practice some form of implemented feedback in the documentation application might result in higher agreement.

Additionally, no prior training of observers regarding pathology detection or scoring occurred. Even though only experienced observers participated, pattern recognition training might have potentially higher agreement among observers in our study.

We used a POCUS system with lower image quality compared to high-end systems. Nevertheless, POCUS systems are used daily in clinical routine bed-side and all observers confirmed that image quality was sufficient for evaluation. Post-hoc evaluation of cine-loops does not allow adaption of planes by the investigator and therefore might not be transferable to the bedside situation, where the examiner can optimize the area of interest. Passing this scenario, all images were acquired by experienced examiners using a standardized protocol, which is representative for image interpretation during clinical routine.

While not directly affecting inter- and intraobserver variability, one has to consider that particularly the posterior paravertebral scanning positions as proposed by Soldati et al. are important for single patient evaluation^17,25^.

Duration of analysis may have been overestimated, if observers did not rate instantly but were distracted from the tool; we thus used a cut-off value of 90 s for maximal time for a single loop.

## Conclusion

Although LUS is a standardized imaging technique, interpretation of specific lung findings in COVID-19 patients reached only fair to moderate interobserver agreement and moderate to substantial intraobserver agreement among a high number of observers. Agreement was highest for more distinct LUS findings such as *air bronchograms* and *subpleural consolidations* and more severe LUS scores*.*

We conclude that in regard to LUS performed for disease monitoring focus on distinct COVID-19-associated LUS findings may be more suitable for disease monitoring, especially in follow-up of patients by more than one investigator.

Considering aforementioned discussed studies and results, a training of observers might pose beneficial effects on agreement and clinical feasibility. Furthermore, we suggest that training material used for LUS in POCUS should pay refined attention to areas such as *B-line* quantification and differentiation of intermediate LUS scores, which revealed only mediocre inter- and intraobserver agreement in our study.

## Supplementary Information

Supplementary Information.

## Abbreviations

ARDS: Acute respiratory distress syndrome
CT: Computed tomography
COVID-19: Coronavirus Disease 2019
ICS: Intercostal space
ICU: Intensive care unit
IQR: Interquartile Range
LUS: Lung ultrasound
PCR: Polymerase chain reaction
POCUS: Point-of-care ultrasound
SARS-CoV-2: Severe Acute Respiratory Syndrome Coronavirus 2
US: Ultrasound
