# Supplementary material for: Point-of-care lung ultrasound in COVID-19 patients: inter- and intra-observer agreement in a prospective observational study
Source: Sci Rep. 2021 May 21;11:10678. doi: 10.1038/s41598-021-90153-2 (PMC8139973; doi:10.1038/s41598-021-90153-2)
Supplement: Supplementary file 1 — Supplementary Information. [file 41598_2021_90153_MOESM1_ESM.docx]

**Point-of-Care Lung Ultrasound in COVID-19 Patients: Inter- and Intraobserver Agreement in a Prospective Observational Study**

Markus H. Lerchbaumer^1*^, Jonathan H. Lauryn^2,3*^, Ulrike Bachmann^4^, Philipp Enghard^5^, Thomas Fischer^1^, Jana Grune^2,3,6^, Niklas Hegemann^2,3^, Dmytro Khadzhynov^5^, Jan Matthias Kruse^5^, Lukas J. Lehner^5^, Tobias Lindner^4^, Timur Oezkan^4^, Daniel Zickler^5^, Wolfgang M. Kuebler^2,3,7,8^, Bernd Hamm^1^, Kai-Uwe Eckardt^5^, Frédéric Muench^5^

** shared first authorship*

^1^ Charité - Universitätsmedizin Berlin, corporate member of Freie Universität Berlin, Humboldt-Universität zu Berlin, Department of Radiology, Berlin, Germany.

^2^ Charité - Universitätsmedizin Berlin, corporate member of Freie Universität Berlin, Humboldt-Universität zu Berlin, Institute of Physiology, Berlin, Germany.

^3^ German Center for Cardiovascular Research (DZHK), partner site Berlin, Germany.
^4^ Department of Emergency Medicine (CVK, CCM), Charité - Universitaetsmedizin, Berlin, Germany.

^5^ Department of Nephrology and Medical Intensive Care, Charité - Universitaetsmedizin, Berlin, Germany.

^6^ Center for Systems Biology, Massachusetts General Hospital Research Institute, Harvard Medical School, Boston.

^7^ The Keenan Research Centre for Biomedical Science at St. Michael´s, Toronto, Canada.
^8^ Departments of Surgery and Physiology, University of Toronto, Toronto, Canada.

Corresponding author:

Frédéric Muench, MD

Department of Nephrology and Medical Intensive Care

Charité - Universitaetsmedizin Berlin
Charitéplatz 1

10117 Berlin

Germany

Tel: +49(0)30 450 614644

Fax: +49(0)30 450 7557901

Email: frederic.muench@charite.de

**Supplementary methods:**

Further statistical analysis included Cochran’s Q test with posthoc McNemar’s test and Bonferroni-corrected p-values related-samples Friedman's two-way analysis of variance by ranks summary, and posthoc Wilcoxon signed-rank test with Bonferroni-adapted p-values.

**Supplementary results:**

***Inter- and intraobserver variability***

Interobserver variability between observers in LUS Score (overall score and individual scores) as well as single pathology rating in all instances (instance 1 - instance 4) resulted in Fleiss’ kappa values of mostly moderate and sometimes fair agreement (**Figure 3A; Table 3s**).

Overall LUS Score and individual LUS scores remained the same over time, except for the individual LUS Score category 3. All scores exhibited moderate agreement between observers, except for LUS score 1 (Instance 1 vs. Instance 4: Overall LUS-Score κ = 0.41 CI:0.39-0.43 vs. κ = 0.43 CI:0.4-0.44; LUS Score 0 κ = 0.53, CI: 0.5-0.56 vs. κ = 0.53, CI: 0.5-0.56; LUS Score 1 κ = 0.32, CI: 0.29-0.34 vs κ = 0.27, CI: 0.24-0.3; LUS Score 2 κ = 0.38, CI 0.35-0.41 vs. κ = 0.38, CI 0.35-0.41; LUS Score 3 κ = 0.45 CI: 0.42-48 vs. κ = 0.59 CI: 0.56-62).

While the agreement on detection of no pathology, pleural thickening and confluent B-Lines exhibited no difference over time, the agreement on the detection of single B-Lines decreased and the agreement on subpleural consolidations as well as air bronchograms increased (Instance 1 vs. Instance 4: No Pathology κ = 0.49, CI: 0.46-0.52 vs. κ = 0.47, CI: 0.44-0.5; Pleural Thickening κ = 0.44, CI: 0.41-0.47 vs.κ = 0.44, CI: 0.41-0.47; Single B-Lines κ = 0.35, CI: 0.32-0.38 vs. κ = 0.22, CI: 0.19-0.25; Confluent B-Lines κ = 0.46 , CI: 0.43-0.49 vs. κ = 0.48, CI: 0.5-0.51; Subpleural consolidations κ = 0.49, CI: 0.46-0.52 vs. κ = 0.59, CI: 0.56-0.62; air bronchograms κ = 0.38, CI: 0.35-0.41 vs. κ = 0.59, CI: 0.56-0.62).

As presented in **Figure 3B and Table 4s,** intraobserver variability in LUS scoring ranged from moderate to substantial agreement (Median κ (IQR = Q1-Q3): Overall LUS score = 0.63 (0.54-0.69); LUS Score 0 = 0.71 (0.6-0.76); LUS Score 1 = 0.52 (0.46-0.58); LUS Score 2 = 0.65 (0.53-0.7); LUS Score 3 = 0.79 (0.74-0.83); No Pathology = 0.65 (0.5-0.78); Pleural Thickening = 0.66 (0.59-0.69); Single B-Lines = 0.49 (0.44-0.53); Confluent B-Lines = 0.55 (0.49-0.64); Subpleural consolidations = 0.67 (0.63-0.76); Air bronchograms = 0.72 (0.56-0.75).

**Group comparisons: LUS-Scores**

Comparison between Observer 1-10 via Kruskal-Wallis-Test revealed a significant difference in the distribution of LUS-Scores in all instances between observer groups (Instance 1: Kruskal-Wallis-H = 16.553, p<.005; Instance 2: Kruskal-Wallis-H = 21,293, p<.005; Instance 3: Kruskal-Wallis-H = 15,055, p<.005; Instance 4: Kruskal-Wallis-H = 17,158, p<.005).

Post-hoc testing with pairwise comparisons (Dunn-Tests with Bonferroni-adjusted p-values) exposed significant differences between Emergency Medicine and Physiology in the first instance and differences in Intensive Care and Physiology scoring in all instances, as well as differences between Intensive Care and Emergency medicine in the third instance. No significantly different distribution of each group compared to correct answers was reported.

(Instance 1: Emergency medicine vs. Physiology: z-score = -3.922, p<.005; Intensive Care vs. Physiology: z-score = -3.009, p<.05. Instance 2: Intensive Care vs. Physiology z-score = -4.614, p<.0005. Instance 3: Intensive Care vs. Physiology z-score = -3.865, p<.05; Intensive Care vs. Emergency medicine z-score: -1.960, p = .05. Instance 4: Intensive Care vs. Physiology z-score = -4.114, p<.0005)

**Group comparison: Detecting lung pathologies in LUS**

A Pearson Chi-Square test was carried out to examine the relation between observer groups and the correct detection of no pathology. The difference between these variables was not significant, concluding there was no difference between observer groups in detecting no pathology (Observer 1-10 - Instance 1: X^2^ (3, N = 1100) = 4.3482, p = .2262; Instance 2: X^2^ (3, N = 1100) = 4.3937, p = .222; Instance 3: X^2^ (3, N = 1100) = 1.1856, p = .7565; Instance 4: X^2^ (3, N = 1100) = 1.7601, p = .6237).

A Pearson Chi-Square test was carried out to examine the relation between observer groups and the correct detection of pleural thickening. The difference between these variables was significant in all 4 instances, concluding there was a significant difference between observer groups in detecting pleural thickening.

(Observer 1-10 - Instance 1: X^2^ (3, N = 1100) = 17.007, p = .0007; Instance 2: X^2^ (3, N = 1100) = 26.1428, p = .0005; Instance 3: X^2^ (3, N = 1100) = 34.7127, p = .0005; Instance 4: X^2^ (3, N = 1100) = 27.2813, p = .0005)

A post-hoc χ2-corrected residues-Test with Bonferroni-adapted p-values (Bonferroni-corrected: p = .00625) was carried out for specifying significant differences between observed and expected frequencies in observer groups further helping to elucidate on differences between observer groups. In the analysis all observed Intensive Care response frequencies from instance 2 to 4 differed significantly from the expected response frequencies for pathology detection (Intensive Care Observer group: Instance 2 adjusted residual = -3.0635, post hoc p-value <.005; Instance 3 adjusted residual = -4.5111 post hoc p-value <.000005; Instance 4 adjusted residual = -4.2758, post-hoc p-value <.00005).

Accordingly, the Intensive Care observer group differed significantly from other observer groups and the correct answer for the detection of pleural thickening in instance 2-4.

A Pearson Chi-Square test was carried out to examine the relation between observer groups and the correct detection of single B-Lines. The difference between these variables was significant in instance 4, concluding there was a significant difference between observer groups in detecting single B-Lines (Observer 1-10 - Instance 4: X^2^ (3, N = 1100) = 14.7097, p = .0005).

A post-hoc χ2-corrected residues-Test with Bonferroni-adapted p-values (Bonferroni-corrected: p = .00625) was carried out for specifying significant differences between observed and expected frequencies in observer groups further helping to elucidate on differences between observer groups. In the analysis of all observers, the observed Emergency medicine response frequencies from instance 4 differed significantly from the expected response frequencies for pathology detection (Emergency Medicine Observer group: Instance 4 adjusted residual = -3.126, post hoc p-value <.005). Accordingly, the Emergency medicine observer group differed significantly from other observer groups and the correct answer for the detection of single-B-Lines in instance 4, hence worsening over time in their detection rate of single-B-Lines.

A Pearson Chi-Square test was carried out to examine the relation between observer groups and the correct detection of confluent B-Lines. The difference between these variables was significant in instances 3 and 4, concluding there was a significant difference between observer groups in detecting confluent B-Lines (Observer 1-10 - Instance 1: X^2^ (3, N = 1100) = 12.926, p = .005; Instance 2: X^2^ (3, N = 1100) = 20.3319, p = .0001; Instance 3: X^2^ (3, N = 1100) = 18.6646, p = .0003; Instance 4: X^2^ (3, N = 1100) = 20.2684, p = .0001)

A post-hoc χ2-corrected residues-Test with Bonferroni-adapted p-values (Bonferroni-corrected: p = .00625) was carried out for specifying significant differences between observed and expected frequencies in observer groups further helping to elucidate on differences between observer groups. In the observed Emergency medicine response frequencies from instance 3 and 4 differed significantly from the expected and observed response frequencies for confluent B-Line detection (Emergency Medicine Observer group: Instance 3: adjusted residual = 2.9518, post hoc p-value <.005; Instance 4: adjusted residual = 3.3923, post hoc p-value <.005).

Accordingly, the Emergency medicine observer group differed significantly from other observer groups and the correct answer for the detection of confluent-B-Lines in instances 3 and 4.

A Pearson Chi-Square test was carried out to examine the relation between observer groups and the correct detection of subpleural consolidations. The difference between these variables was not significant, concluding there was no difference between observer groups in detecting subpleural consolidations.

(Observer 1-10 - Instance 1: X^2^ (3, N = 1100) =1.7804, p = .6192; Instance 2: X^2^ (3, N = 1100) =0.9427, p = .8151; Instance 3: X^2^ (3, N = 1100) = 0.17, p = .9823; Instance 4: X^2^ (3, N = 1100) = 0.5677, p = .9038)

A Pearson Chi-Square test was carried out to examine the relation between observer groups and the correct detection of air bronchograms. The difference between these variables was significant in instances 1-4, concluding there was a significant difference between observer groups in detecting air bronchograms in lung ultrasound video loops (Observer 1-10 - Instance 1: X^2^ (3, N = 1100) = 44.5191, p <.0005 .; Instance 2: X^2^ (3, N = 1100) = 39.6625, p <.0005; Instance 3: X^2^ (3, N = 1100) = 24.1463, p <.0005 .; Instance 4: X^2^ (3, N = 1100) = 14.4874, p <.005).

A post-hoc χ2-corrected residues-Test with Bonferroni-adapted p-values (Bonferroni-corrected: p = .00625) was carried out for specifying significant differences between observed and expected frequencies in observer groups further helping to elucidate on differences between observer groups. In the analysis of the observed response frequencies in instance 1 all observer groups differed significantly, while in instance 2 to 4 only Emergency Medicine and Physiology observers differed significantly from the expected and observed response frequencies for air bronchogram detection (Intensive Care Observer group - Instance 1: adjusted residual = -2.9055; post hoc Test p-value <.005; Emergency Medicine Observer group - Instance 1: adjusted residual = -3.4449, post hoc Test p-value <.0005; Instance 2: adjusted residual =-4.2275, post hoc p-value <.00005; Instance 3: adjusted residual = -3.6204, post hoc p-value <.0005; Instance 4: adjusted residual = -3.4668, post hoc p-value <.005; Physiology Observer group - Instance 1: adjusted residual = 6.4749 ;post hoc Test p-value <.000005; Instance 2: adjusted residual =5.8379, post hoc p-value <.000005; Instance 3: adjusted residual = 4.423, post hoc p-value <.00005; Instance 4: adjusted residual = 2.7552, post hoc p-value <.05).

Concluding differences in response frequencies, the Intensive care observer group was able to reach greater consensus with the correct answers over time or over viewing instances. While the Emergency Medicine observer group exhibited continuous differences in response frequencies, the Physiology observer group saw some form of improvement concerning response frequency differences to correct answers over time, seen in the decrease of adjusted residuals from instance 1 to 4.

**Intraobserver learning effect over time for single pathologies**

We used Cochrane’s-Q-Test for determining intraobserver differences over 4 viewing instances (= #1, #2, #3, #4) and the correct answer (= C), hypothesizing potential learning effects in single observers over time.

Concerning the learning curve in detecting no pathology Cochran's-Q-Test did not indicate any differences among the 5 instances, including the correct answer in Observer 1 (χ2(4) = 7.512, p = .111), Observer 2 (χ2(4) =2.556, p = .635), Observer 7(χ2(4) =6.000, p = .199), Observer 9(χ2(4) = 6.4, p = .171) and Observer 10(χ2(4) =4.947, p = .293) resulting in the retainment of the null hypothesis.

Cochran's-Q-Test indicated differences among the 5 instances, including the correct answer in Observer 3 (χ2(4) =16.711, p = .002,), Observer 4(χ2(4) =14.444, p = .006), Observer 5(χ2(4) = 17.25, p = .002), Observer 6(χ2(4) = 28.143 , p = .0005) and Observer 8 (χ2(4) = 13.511, p = .009), resulting in the rejection of the null hypothesis. We tested via multiple McNemar’s tests with Bonferroni correction for unfolding specific significant differences over time.

Observer 3 showed significant differences to correct answer in Instances1 and 2 (#1 vs. C: p<.05; #2 vs. C: p<.005). Observer 4 revealed significant difference between Instance 4 and correct answer, (#4 vs. C: p <.005). Observer 5 revealed significant differences between Instance 3, 4 and correct answers. (#4 vs. C: p<.005, #3 vs. C: p<.05). Observer 6 exhibited significant differences between Instance 1, 2, 3, 4 and correct answer (#1 vs. C: p<.005; #2 vs. C: p<.005; #3 vs. C: p<.005; #4 vs. C: p<.005). Observer 8 displayed differences between Instance 2, 3, 4 and correct answer, as well as instance 1, which did not come below Bonferroni-corrected significance (#2 vs. C: p= .077; #3 vs. C: p=.077; #4 vs. C: p=.077; #2 vs. #1: p=.455; #3 vs. #1: p=.455; #4 vs. #1: p=.455).

Concerning the learning curve in detecting Pleural thickening Cochran's-Q-Test did not indicate any differences among the 5 instances, including the correct answer in Observer 2 (χ2(4) =9.179, p = .057), Observer 5 (χ2(4) =1.524, p = .822), Observer 8 (χ2(4) =6.677, p = .154) resulting in the retainment of the null hypothesis.

Cochran's-Q-Test indicated differences among the 5 instances, including the correct answer in Observer 1 (χ2(4) =19.725, p<.005), Observer 3(χ2(4) = 19.182, p<.005), Observer 4(χ2(4) = 41,299, p<.0005), Observer 6(χ2(4) =26.796, p <.0005), Observer 7 (χ2(4) =16.444, p<.005), Observer 9 (χ2(4) = 84.833, p<.0005) and Observer 10 (χ2(4) = 18.927 , p<.005) resulting in the rejection of the null hypothesis. We tested via multiple McNemar’s tests with Bonferroni correction for unfolding specific significant differences over time (Observer 1: #3 vs. #1 p<.05, #3 vs. C p<.005; Observer 3: #1 vs. #3 p<.05, #1 vs. C p<.005, #4 vs. C p<.05; Observer 4: #1 vs. C p<.005, #2 vs. C p<.005, #3 vs. C p<.005, #4 vs. C p<.005; Observer 6: #1 vs. C p<.005, #2 vs. C p<.005, #3 vs. C p<.05, #4 vs. C p<.05; Observer 7: #1 vs. C p<.005, #2 vs. C p<.05; Observer 9: #1 vs. C p<.005, #2 vs. C p<.005, #3 vs. C p<.005, #4 vs. C p<.005; Observer 10: #1 vs. C p<.005, #2 vs. C p<.005, #4 vs. C p<.05).

Concerning the learning curve in detecting single B-Lines Cochran's-Q-Test did not indicate any differences among the 5 instances, including the correct answer in Observer 1 (χ2(4)=1.963, p=.743), Observer 4 (χ2(4)=7.556, p=.109), Observer 10 (χ2(4)=9.223, p=.056) resulting in the retainment of the null hypothesis.

Cochran's-Q-Test indicated differences among the 5 instances, including the correct answer in Observer 2 (χ2(4)=16.206, p<.005), Observer 3 (χ2(4)=12.709, p<.05), Observer 5(χ2(4)=21.613, p<.005), Observer 6(χ2(4)=11.966, p<.05), Observer 7 (χ2(4) =18.667, p<.005), Observer 8 (χ2(4)=17.358, p<.005) and Observer 9 (χ2(4) =14.447, p<.05) resulting in the rejection of the null hypothesis and multiple McNemar’s tests with Bonferroni correction for unfolding specific significant differences over time (Observer 2: #4 vs. #1 p<.005; Observer 3: #4 vs. C p<.05; Observer 5: #4 vs C p<.005, #2 vs. C p<.005, #1 vs. C p<.05; Observer 6: #4 vs. C p<.05; Observer 7: #2 vs. C p<.05, #3 vs. C p<.05, #4 vs. C p<.005; Observer 8: #2 vs. C p<.005, #3 vs. C p<.005, #4 vs. C p<.005; Observer 9: #2 vs. #4 p<.05).

Concerning the learning curve in detecting confluent B-Lines Cochran's-Q-Test did not indicate any differences among the 5 instances, including the correct answer in Observer 1 (χ2(4)=1.854, p=.763), Observer 2 (χ2(4)=6.850, p=.144) and Observer 4 (χ2(4)=6.066, p=.194) resulting in the retainment of the null hypothesis.

Cochran's-Q-Test indicated differences among the 5 instances, including the correct answer in Observer 3 (χ2(4) = 15.556, p<.005), Observer 5 (χ2(4) = 66.955, p<.005), Observer 6 (χ2(4)= 65.841, p<.005), Observer 7(χ2(4) =28.951, p<.005), Observer 8 (χ2(4) =47.462, p<.005), Observer 9 (χ2(4)=46.716, p<.005) and Observer 10 (χ2(4) =23.63, p<.005) resulting in the rejection of the null hypothesis and multiple McNemar’s tests with Bonferroni correction for unfolding specific significant differences over time (Observer 3: #1 vs #3 p<.05, #1 vs. #4 p<.05, #1 vs. C p<.005; Observer 5: #2 vs. #1 p<.005, #3 vs. #1 p<.005, #4 vs. #1 p<.005, #2 vs. C p<.005, #3 vs. C p<.005, #4 vs C p<.005; Observer 6: #1 vs. C p<.005, #2 vs. C p<.005, #3 vs. C p<.005, #4 vs. C p<.005, #1 vs. #2 p<.005, #1 vs. #3 p<.005, #1 vs. #4 p<.05;

Observer 7: #1 vs. C p<.005, #4 vs. C p<.05, #1 vs. #2 p<.05; Observer 8: #1 vs. C p<.005, #2 vs. C p<.005, #3 vs. C p<.005, #4 vs. C p<.005; Observer 9: #1 vs. C p<.005, #2 vs. C p<.05, #4 vs. C p<.005, #1 vs. #2 p<.05, #1 vs. #3 p<.005, #1 vs. #4 p<.05; Observer 10: #1 vs #4 p<.05, #2 vs. #4 p<.05, #1 vs. C p<.005, #2 vs. C p<.005).

Concerning the learning curve in detecting subpleural consolidations Cochran's-Q-Test did not indicate any differences among the 5 instances, including the correct answer in Observer 1 (χ2(4)=4.068, p=.397), Observer 2 (χ2(4)=2.933, p=.569), Observer 3 (χ2(4)=1.827, p=.768), Observer 4 (χ2(4)=3.15, p=.533), Observer 5 (χ2(4)=1.612, p=.807), Observer 6 (χ2(4)=1.803, p=.772), Observer 8 (χ2(4)=4.26, p=.372) and Observer 9 (χ2(4)=1.333, p=.856) resulting in the retainment of the null hypothesis.

Cochran's-Q-Test indicated differences among the 5 instances, including the correct answer in Observer 7 (χ2(4)=23.719, p<.0005) and Observer 10 (χ2(4)=30.863, p<.0005) resulting in the rejection of the null hypothesis and multiple McNemar’s tests with Bonferroni correction for unfolding specific significant differences over time.

(Observer 7: #1 vs. C p<.005, #2 vs. C p<.0005, #3 vs. C p<.05; Observer 10: #1 vs. #3 p<.05, #1 vs. C p<.005, #2 vs. C p<.005, #4 vs. C p<.05)

Concerning the learning curve in detecting air bronchograms Cochran's-Q-Test did not indicate any differences among the 5 instances, including the correct answer in Observer 2 (χ2(4)=3.172, p=.529), Observer 3 (χ2(4)=4.933, p=.294), Observer 6 (χ2(4)=1.714, p=.788) and Observer 7 (χ2(4)=2.933, p=.569) resulting in the retainment of the null hypothesis.

Cochran's-Q-Test indicated differences among the 5 instances, including the correct answer in Observer 1 (χ2(4)=10.712, p<.05), Observer 4 (χ2(4)=26.47, p<.0005), Observer 5 (χ2(4)=22, p<.0005), Observer 8 (χ2(4)=15.667, p<.005), Observer 9 (χ2(4)=25.217, p<.0005) and Observer 10 (χ2(4)=53.981, p<.0005) resulting in the rejection of the null hypothesis and multiple McNemar’s tests with Bonferroni correction for unfolding specific significant differences over time (Observer 1 after Bonferroni correction: #3 vs. C p=.253, #4 vs. C p=.253, #3 vs. 1 p=.253, #4 vs. #1 p=.253; Observer 4: #1 vs. C p<.05, #2 vs. C p<.0005, #3 vs. C p<.05, #4 vs. C p<.0005; Observer 5: #1 vs. C p<.005, #2 vs. C p<.005, #3 vs. C p=.005, #4 vs. C p<.05; Observer 8: #3 vs. #1 p<.05, #3 vs. #C p<.05; Observer 9: #1 vs. C p<.0005, #2 vs. C p<.005, #3 vs. C p<.05; Observer 10: #1 vs. #3 p<.0005, #1 vs. #4 p<.0005, #2 vs. #4 p<.05, #1 vs. C p<.0005, #2 vs. C p<.0005)

**Intraobserver learning effect over time for LUS scoring**

We investigated with the Friedman-Test whether a difference in scoring can be observed over time, potentially linking to a learning effect. Observer’s specific LUS scoring over 4 instances and the predetermined correct answer were plotted accordingly by rank and frequency.

Determined by the Friedman-Test there was no statistically significant difference in LUS scoring over the time in Observer 1 (χ2(4) = .285, p = 0.991).

The Friedman-Test revealed a statistically significant difference in Observer 2 concerning LUS scoring over the time (χ2(4) = 22.759, p <.0005). A Wilcoxon signed rank test with a Bonferroni correction was carried out as post hoc test for comparison revealing no significant difference in adjusted p-values (Instance 2 vs. correct answer p=.148, without Bonferroni correction p=.015; Instance 3 vs. correct answer p=.253, without Bonferroni correction p=.025)

The Friedman-Test revealed a statistically significant difference in Observer 3 concerning LUS scoring over the time (χ2(4) = 12,681, p <.05). A Wilcoxon signed rank test with a Bonferroni correction was carried out as post hoc test for comparison revealing no significant difference in adjusted p-values.

The Friedman-Test revealed a statistically significant difference in Observer 4 concerning LUS scoring over the time (χ2(4) = 23,256, p <.0005). A Wilcoxon signed rank test with a Bonferroni correction was carried out as post hoc test for comparison revealing no significant difference in adjusted p-values.

Determined by the Friedman-Test there was no statistically significant difference in LUS scoring over the time in Observer 5 (χ2(4) = 7.271, p = 0.122).

The Friedman-Test revealed a statistically significant difference in Observer 6 in LUS scoring over the time (χ2(4) = 16.232, p <.005). A Wilcoxon signed rank test with a Bonferroni correction was carried out as posthoc test for comparison revealing no significant difference in adjusted p-values.

The Friedman-Test revealed a statistically significant difference in LUS scoring over time in Observer 7(χ2(4) = 34,682, p <.0005). A Wilcoxon signed rank test with a Bonferroni correction was carried out as posthoc test for comparison revealing a significant difference in adjusted p-values between Instance 4 and the correct standard (p=.024)

The Friedman-Test revealed a statistically significant difference in LUS scoring over time in Observer 8(χ2(4) = 31.569, p <.0005). A Wilcoxon signed rank test with a Bonferroni correction was carried out as posthoc test for comparison revealing a significant difference in adjusted p-values between instance 1 and 3 (p=.027), and instance 1 and 4 the correct standard (p=.011).

The Friedman-Test revealed a statistically significant difference in LUS scoring over the time in Observer 9 (χ2(4) =13.656, p <.008). A Wilcoxon signed rank test with a Bonferroni correction was carried out as posthoc test for comparison revealing no significant difference in adjusted p-values.

The Friedman-Test revealed a statistically significant difference in LUS scoring over the time in Oberserver 10 (χ2(4) =43.031, p <.0005). A Wilcoxon signed rank test with a Bonferroni correction was carried out as posthoc test for comparison revealing a significant difference in adjusted p-values between instance 1 and 3 (p=0.027) and instance 1 and 4 (p=.009) and instance 1 and the correct answer (p=.006).


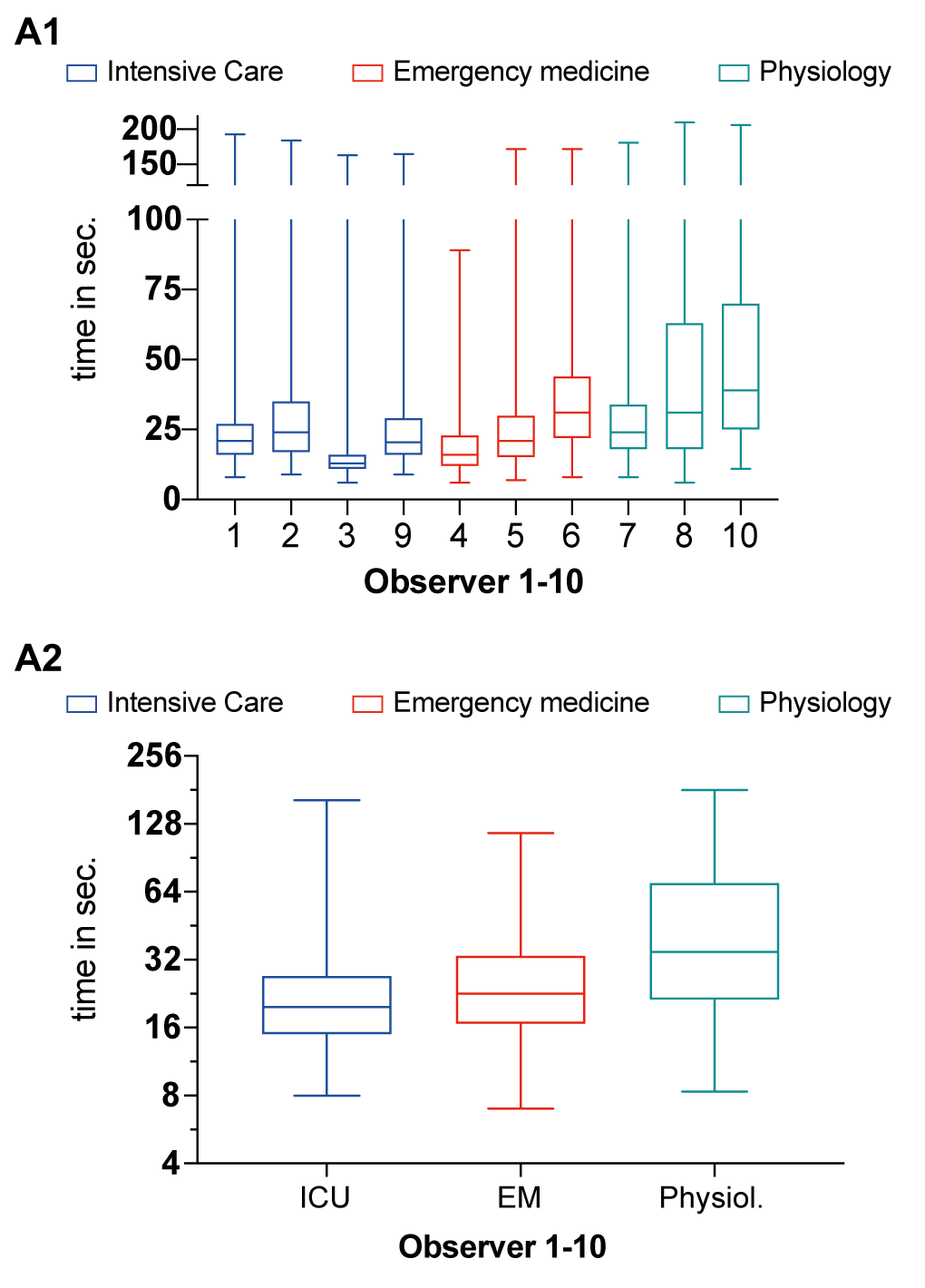

**Figure 1s. Group comparisons: Time Recording Per View**

**A1:**  Graphical representation of median, upper and lower quartile, as well as minimum and maximum of observer time recording per video loop in seconds ordered by group affiliation.

**A2:** After testing for normality, group comparison was carried out via nonparametric One-Way ANOVA, where a significant difference in the distribution of time recording duration between groups was detected (Kruskal-Wallis-H = 227.5, p<.0005). Dunn’s multiple comparison testing with Bonferroni adjusted p-values unveiled a difference in distribution between all observer groups. (Intensive Care vs. Emergency medicine: z-score 7.088, p<.0005; Intensive Care vs. Physiology: z-score 15.07, p<.0005, Emergency medicine vs. Physiology: z-score 7.986, p<.0005) Reported mean in sec ± standard deviation per observer group: Intensive Care = 25.22±19.02; Emergency medicine = 28.38±18.03; Physiology = 48.78±36.04.


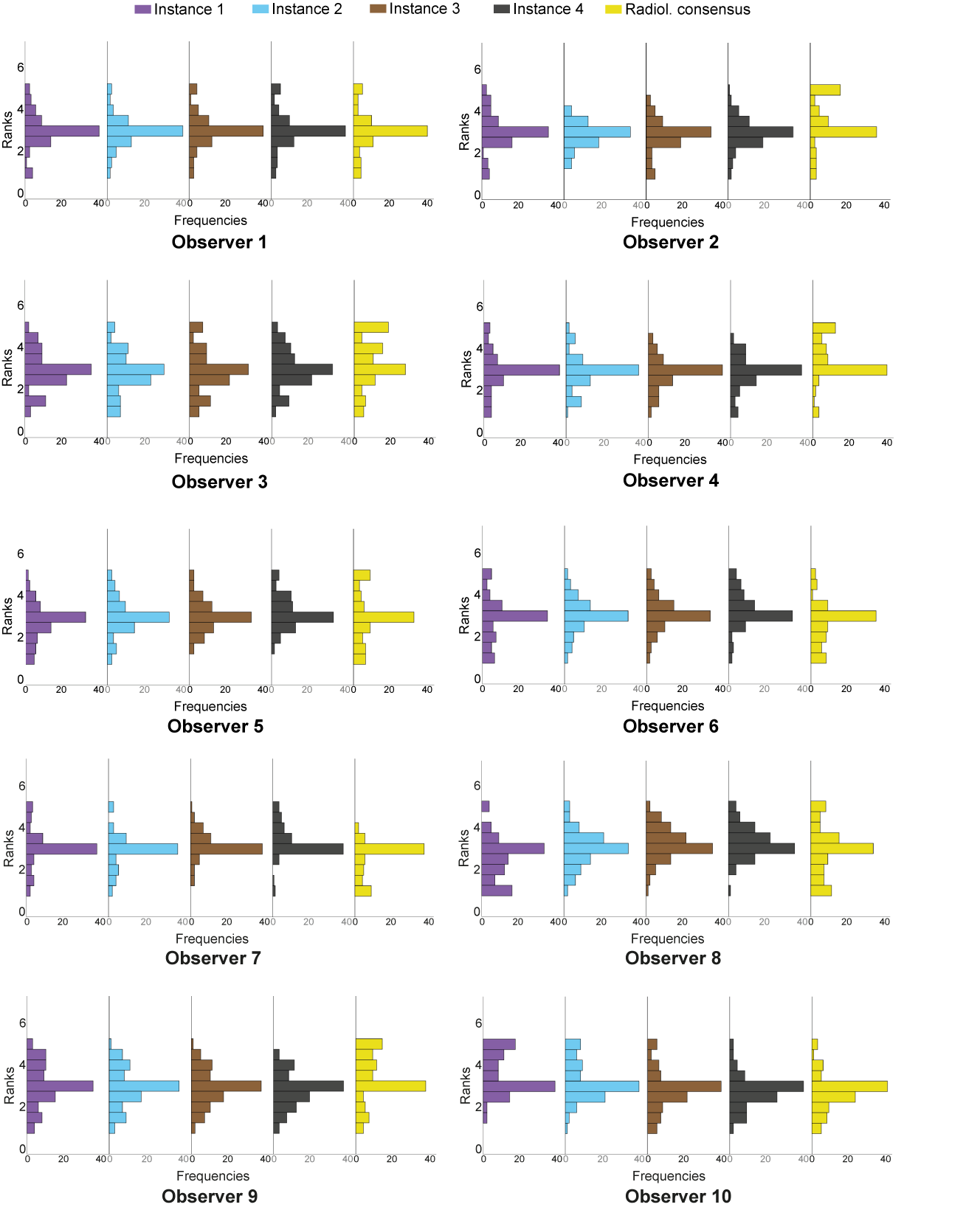


**Figure 2s. Intraobserver learning effect for LUS Score (1)**

We investigated with the Friedman-Test whether a difference in scoring can be observed over time, potentially linking to a learning effect. Observer’s specific LUS scoring over 4 instances and the predetermined correct answer were plotted accordingly by rank and frequency; cf. supplementary results for statistical results

**Table 1s. Patient characteristics**

| **Patient ID** | **L01** | **L02** | | | **L03** | | | **L04** | | | **L05** | | | **L06** | | | **L07** | | | **L08** | | | **L09** | | | **L10** | | | **L11** | | **L12** | **L13** |
| --- | --- | --- | --- | --- | --- | --- | --- | --- | --- | --- | --- | --- | --- | --- | --- | --- | --- | --- | --- | --- | --- | --- | --- | --- | --- | --- | --- | --- | --- | --- | --- | --- |
| **Sex** | m | m | | | m | | | m | | | m | | | m | | | m | | | m | | | m | | | m | | | m | | m | m |
| **Age** | 75 | 69 | | | 61 | | | 62 | | | 78 | | | 78 | | | 47 | | | 66 | | | 66 | | | 77 | | | 86 | | 67 | 49 |
| **Clinical parameters at the time of lung ultrasound examination** | | | | | | | | | | | | | | | | | | | | | | | | | | | | | | | | |
| **Time in the ICU in days** | 45 | 16 | | 5 | | | 19 | | | 31 | | | 42 | | | 11 | | | 11 | | | 10 | | | 1 | | | 1 | | | 33 | 52 |
| **Intubation/Tracheal Cannula** | No | No | | Yes | | | Yes | | | Yes | | | Yes | | | Yes | | | Yes | | | Yes | | | Yes | | | Yes | | | Yes | Yes |
| **BIPAP** | No | No | | Yes | | | Yes | | | No | | | No | | | Yes | | | No | | | Yes | | | Yes | | | No | | | No | Yes |
| **CPAP** | No | No | | No | | | No | | | Yes | | | Yes | | | No | | | Yes | | | No | | | No | | | Yes | | | Yes | No |
| **O2 via nasal cannula** | Yes | Yes | | No | | | No | | | No | | | No | | | No | | | No | | | No | | | No | | | No | | | No | No |
| **vv-ECMO** | No | No | | No | | | No | | | No | | | No | | | Yes | | | No | | | No | | | No | | | No | | | No | Yes |
| **CRRT** | No | No | | Yes | | | Yes | | | No | | | No | | | Yes | | | No | | | Yes | | | Yes | | | No | | | No | Yes |
| **Pre-existing lung conditions in patients** | | | | | | | | | | | | | | | | | | | | | | | | | | | | | | | | |
| **COPD** | No | No | | No | | | No | | | No | | | No | | | No | | | No | | | No | | | No | | | No | | | Yes | No |
| **Lung emphysema** | No | No | | No | | | No | | | No | | | No | | | No | | | No | | | No | | | No | | | No | | | No | No |
| **Lung fibrosis** | No | No | | No | | | No | | | No | | | No | | | No | | | No | | | No | | | No | | | No | | | No | No |
| **Asthma** | No | No | | No | | | No | | | Yes | | | No | | | No | | | No | | | No | | | No | | | No | | | No | No |
| **Laboratory findings on date of examination and peak during hospital admission** | | | | | | | | | | | | | | | | | | | | | | | | | | | | | | | | |
| **CRP (in mg/l)** | 7 | 15 | 173 | | | 110 | | | 115 | | | 46 | | | 114 | | | 187 | | | 315 | | | 99 | | | 50 | | | 19 | | 104 |
| **peak CRP (in mg/l)** | 163 | 189 | 496 | | | 318 | | | 339 | | | 500 | | | 461 | | | 318 | | | 340 | | | 298 | | | 436 | | | 345 | | 333 |
| **PCT (in µg/l)** | 0.9 | 0.5 | 0.6 | | | 2.5 | | | 1.5 | | | 0.5 | | | 6.6 | | | 2 | | | 3.5 | | | 0.4 | | | 0.1 | | | 2 | | 4.3 |
| **peak PCT (in µg/l)** | 5.7 | 0.8 | 1.8 | | | 8.2 | | | 2.6 | | | 30 | | | 6.8 | | | 4.6 | | | 10.9 | | | 2.9 | | | 8.8 | | | 13.6 | | 9.3 |
| **peak D-Dimers (in FEU)** | 11.2 | 2.7 | 5.2 | | | 3 | | | 2 | | | 8.6 | | | 20 | | | 7.2 | | | 9.1 | | | 2.8 | | | 20 | | | 13.6 | | 20 |
| **peak IL-6 (in pg/ml)** | 420 | 689 | 348 | | | 4458 | | | 250 | | | 3862 | | | 550 | | | 588 | | | 3146 | | | 202 | | | 120 | | | 2475 | | 1287 |
| **peak Ferritin (in µg/l)** | n.a. | 1056 | 1164 | | | 2755 | | | 3880 | | | 4100 | | | 3120 | | | 5906 | | | 15280 | | | 324 | | | 1208 | | | 6652 | | 14247 |

**Table 2s. Interobserver Variability for single pathologies**

| **Observer 1-10** | | | | | |
| --- | --- | --- | --- | --- | --- |
|  | Instance | Kappa | p value | Lower 95 Asymptotic CI Bound | Upper 95 Asymptotic CI Bound |
| LUS Score | #1 | 0.409 | <0.0005 | 0.392 | 0.426 |
|  | #2 | 0.401 | <0.0005 | 0.384 | 0.419 |
|  | #3 | 0.428 | <0.0005 | 0.41 | 0.445 |
|  | #4 | 0.425 | <0.0005 | 0.408 | 0.442 |
|  | | | | | |
| No Pathology | #1 | 0.489 | <0.0005 | 0.46 | 0.518 |
|  | #2 | 0.43 | <0.0005 | 0.401 | 0.459 |
|  | #3 | 0.489 | <0.0005 | 0.459 | 0.518 |
|  | #4 | 0.467 | <0.0005 | 0.438 | 0.497 |
|  | | | | | |
| Pleural Thickening | #1 | 0.442 | <0.0005 | 0.413 | 0.471 |
|  | #2 | 0.431 | <0.0005 | 0.402 | 0.461 |
|  | #3 | 0.424 | <0.0005 | 0.395 | 0.454 |
|  | #4 | 0.439 | <0.0005 | 0.41 | 0.468 |
|  | | | | | |
| Single B-Lines | #1 | 0.349 | <0.0005 | 0.32 | 0.378 |
|  | #2 | 0.281 | <0.0005 | 0.252 | 0.31 |
|  | #3 | 0.295 | <0.0005 | 0.266 | 0.325 |
|  | #4 | 0.221 | <0.0005 | 0.192 | 0.25 |
|  | | | | | |
| Confluent B-Lines | #1 | 0.461 | <0.0005 | 0.431 | 0.49 |
|  | #2 | 0.482 | <0.0005 | 0.453 | 0.511 |
|  | #3 | 0.435 | <0.0005 | 0.406 | 0.465 |
|  | #4 | 0.481 | <0.0005 | 0.452 | 0.51 |
|  | | | | | |
| Subpleural Consolidations | #1 | 0.488 | <0.0005 | 0.458 | 0.517 |
|  | #2 | 0.442 | <0.0005 | 0.413 | 0.471 |
|  | #3 | 0.497 | <0.0005 | 0.468 | 0.526 |
|  | #4 | 0.59 | <0.0005 | 0.561 | 0.619 |
|  | | | | | |
| Air bronchograms | #1 | 0.38 | <0.0005 | 0.35 | 0.409 |
|  | #2 | 0.451 | <0.0005 | 0.422 | 0.48 |
|  | #3 | 0.514 | <0.0005 | 0.485 | 0.544 |
|  | #4 | 0.589 | <0.0005 | 0.56 | 0.619 |

**Table 3s. Interobserver variability for LUS score categories**

| **Observer 1-10** | |  |  |  |  |  |
| --- | --- | --- | --- | --- | --- | --- |
| **Instance** | **Rating Category LUS Score** | **Conditional probability** | **Kappa** | **p value** | **Lower 95 Asymptotic CI Bound** | **Upper 95 Asymptotic CI Bound** |
| #1 | 0 | 0.631 | 0.527 | 0.000 | 0.498 | 0.556 |
|  | 1 | 0.543 | 0.315 | 0.000 | 0.286 | 0.344 |
|  | 2 | 0.559 | 0.383 | 0.000 | 0.354 | 0.413 |
|  | 3 | 0.54 | 0.451 | 0.000 | 0.422 | 0.481 |
|  | | | | | | |
| #2 | 0 | 0.626 | 0.518 | 0.000 | 0.489 | 0.548 |
|  | 1 | 0.513 | 0.284 | 0.000 | 0.255 | 0.313 |
|  | 2 | 0.553 | 0.356 | 0.000 | 0.327 | 0.386 |
|  | 3 | 0.59 | 0.517 | 0.000 | 0.488 | 0.546 |
|  | | | | | | |
| #3 | 0 | 0.629 | 0.515 | 0.000 | 0.486 | 0.544 |
|  | 1 | 0.526 | 0.321 | 0.000 | 0.292 | 0.35 |
|  | 2 | 0.582 | 0.394 | 0.000 | 0.365 | 0.423 |
|  | 3 | 0.607 | 0.536 | 0.000 | 0.507 | 0.565 |
|  | | | | | | |
| #4 | 0 | 0.634 | 0.531 | 0.000 | 0.502 | 0.56 |
|  | 1 | 0.497 | 0.273 | 0.000 | 0.244 | 0.303 |
|  | 2 | 0.574 | 0.383 | 0.000 | 0.354 | 0.413 |
|  | 3 | 0.66 | 0.594 | 0.000 | 0.564 | 0.623 |

**Table 4s. Intraobserver Variability**

|  | **Observer** | **Kappa** | **p value** | **Lower 95 Asymptotic CI Bound** | **Upper 95 Asymptotic CI Bound** |
| --- | --- | --- | --- | --- | --- |
| LUS Score | 1 | 0.683 | <0.0005 | 0.636 | 0.731 |
|  | 2 | 0.745 | <0.0005 | 0.697 | 0.793 |
|  | 3 | 0.49 | <0.0005 | 0.44 | 0.54 |
|  | 4 | 0.686 | <0.0005 | 0.638 | 0.733 |
|  | 5 | 0.637 | <0.0005 | 0.586 | 0.688 |
|  | 6 | 0.631 | <0.0005 | 0.584 | 0.677 |
|  | 7 | 0.697 | <0.0005 | 0.65 | 0.744 |
|  | 8 | 0.538 | <0.0005 | 0.485 | 0.591 |
|  | 9 | 0.598 | <0.0005 | 0.549 | 0.647 |
|  | 10 | 0.547 | <0.0005 | 0.501 | 0.593 |
|  | | | | | |
| No Pathology | 1 | 0.612 | <0.0005 | 0.532 | 0.692 |
|  | 2 | 0.768 | <0.0005 | 0.688 | 0.848 |
|  | 3 | 0.37 | <0.0005 | 0.29 | 0.45 |
|  | 4 | 0.816 | <0.0005 | 0.736 | 0.896 |
|  | 5 | 0.393 | <0.0005 | 0.313 | 0.473 |
|  | 6 | 0.715 | <0.0005 | 0.635 | 0.795 |
|  | 7 | 0.812 | <0.0005 | 0.732 | 0.892 |
|  | 8 | 0.529 | <0.0005 | 0.449 | 0.609 |
|  | 9 | 0.574 | <0.0005 | 0.494 | 0.654 |
|  | 10 | 0.678 | <0.0005 | 0.598 | 0.758 |
|  | | | | | |
| Pleural Thickening | 1 | 0.61 | <0.0005 | 0.53 | 0.69 |
|  | 2 | 0.632 | <0.0005 | 0.552 | 0.712 |
|  | 3 | 0.46 | <0.0005 | 0.38 | 0.54 |
|  | 4 | 0.665 | <0.0005 | 0.585 | 0.745 |
|  | 5 | 0.666 | <0.0005 | 0.586 | 0.746 |
|  | 6 | 0.679 | <0.0005 | 0.599 | 0.759 |
|  | 7 | 0.745 | <0.0005 | 0.665 | 0.825 |
|  | 8 | 0.548 | <0.0005 | 0.468 | 0.628 |
|  | 9 | 0.658 | <0.0005 | 0.578 | 0.738 |
|  | 10 | 0.704 | <0.0005 | 0.624 | 0.784 |
|  | | | | | |
| Single B-Lines | 1 | 0.518 | <0.0005 | 0.438 | 0.598 |
|  | 2 | 0.573 | <0.0005 | 0.493 | 0.653 |
|  | 3 | 0.451 | <0.0005 | 0.371 | 0.531 |
|  | 4 | 0.507 | <0.0005 | 0.427 | 0.587 |
|  | 5 | 0.222 | <0.0005 | 0.142 | 0.302 |
|  | 6 | 0.487 | <0.0005 | 0.407 | 0.567 |
|  | 7 | 0.588 | <0.0005 | 0.508 | 0.668 |
|  | 8 | 0.403 | <0.0005 | 0.323 | 0.483 |
|  | 9 | 0.495 | <0.0005 | 0.415 | 0.575 |
|  | 10 | 0.452 | <0.0005 | 0.372 | 0.532 |
|  | | | | | |
| Confluent B-Lines | 1 | 0.694 | <0.0005 | 0.614 | 0.774 |
|  | 2 | 0.671 | <0.0005 | 0.591 | 0.751 |
|  | 3 | 0.458 | <0.0005 | 0.378 | 0.538 |
|  | 4 | 0.629 | <0.0005 | 0.549 | 0.709 |
|  | 5 | 0.433 | <0.0005 | 0.353 | 0.513 |
|  | 6 | 0.57 | <0.0005 | 0.49 | 0.65 |
|  | 7 | 0.495 | <0.0005 | 0.415 | 0.575 |
|  | 8 | 0.498 | <0.0005 | 0.418 | 0.578 |
|  | 9 | 0.579 | <0.0005 | 0.499 | 0.659 |
|  | 10 | 0.522 | <0.0005 | 0.442 | 0.602 |
|  | | | | | |
| Subpleural consolidations | 1 | 0.811 | <0.0005 | 0.731 | 0.891 |
|  | 2 | 0.895 | <0.0005 | 0.815 | 0.975 |
|  | 3 | 0.52 | <0.0005 | 0.44 | 0.6 |
|  | 4 | 0.74 | <0.0005 | 0.659 | 0.82 |
|  | 5 | 0.642 | <0.0005 | 0.562 | 0.722 |
|  | 6 | 0.718 | <0.0005 | 0.638 | 0.798 |
|  | 7 | 0.648 | <0.0005 | 0.568 | 0.728 |
|  | 8 | 0.671 | <0.0005 | 0.591 | 0.751 |
|  | 9 | 0.664 | <0.0005 | 0.584 | 0.744 |
|  | 10 | 0.604 | <0.0005 | 0.524 | 0.684 |
|  | | | | | |
| Air bronchograms | 1 | 0.815 | <0.0005 | 0.735 | 0.895 |
|  | 2 | 0.729 | <0.0005 | 0.649 | 0.809 |
|  | 3 | 0.674 | <0.0005 | 0.594 | 0.755 |
|  | 4 | 0.392 | <0.0005 | 0.312 | 0.472 |
|  | 5 | 0.708 | <0.0005 | 0.628 | 0.788 |
|  | 6 | 0.735 | <0.0005 | 0.655 | 0.815 |
|  | 7 | 0.748 | <0.0005 | 0.668 | 0.828 |
|  | 8 | 0.603 | <0.0005 | 0.523 | 0.683 |
|  | 9 | 0.781 | <0.0005 | 0.701 | 0.861 |
|  | 10 | 0.437 | <0.0005 | 0.356 | 0.517 |

**
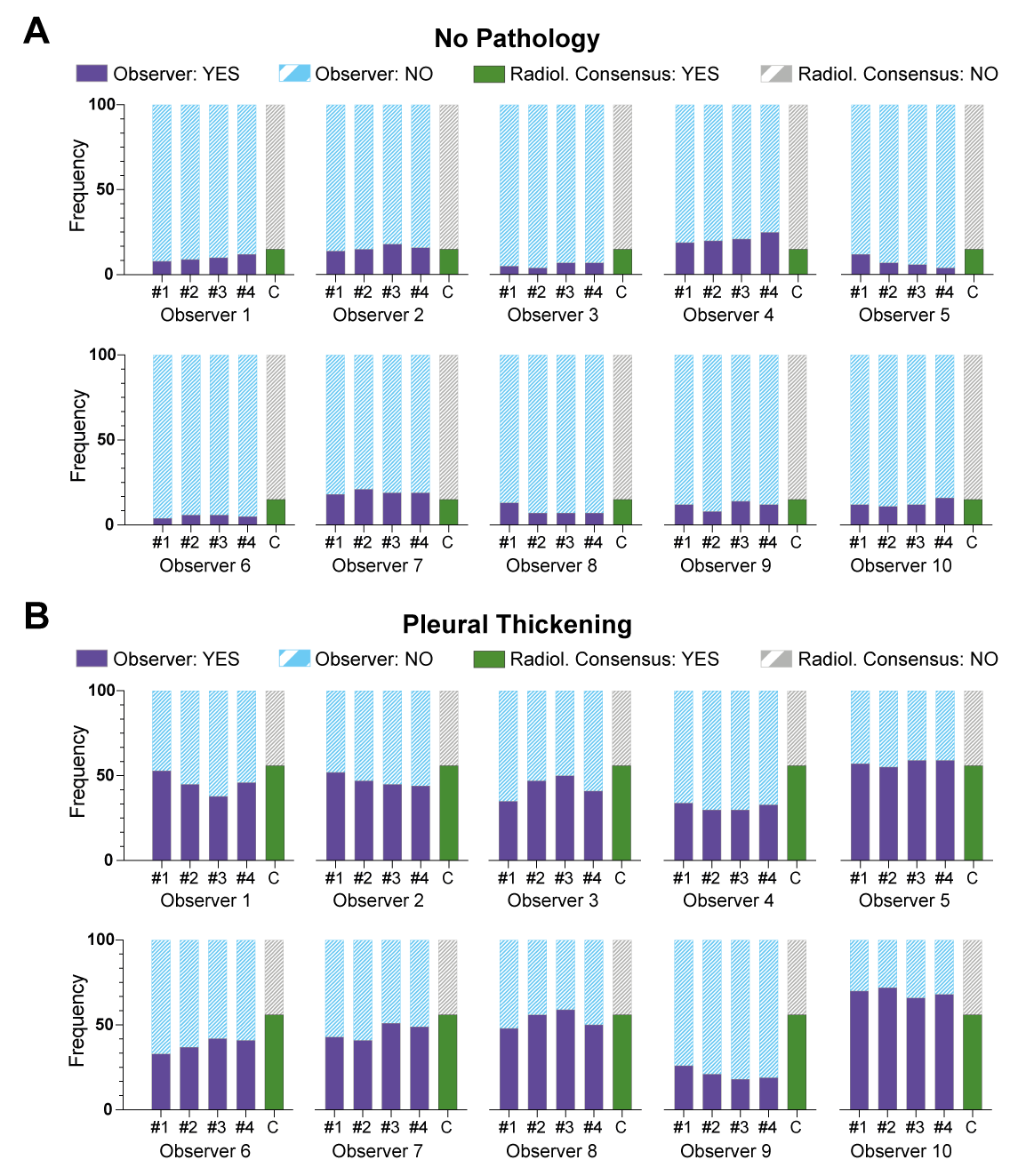
** **Figure 3-1s.** **Intra-observer learning effect over time**. Graphic representation of count of binary response frequency for specific pathologies in each 100 video loops per instance and observer. We used Cochrane’s-Q-Test for determining intraobserver differences over 4 viewing instances (= #1, #2, #3, #4) and the answer determined by radiological consensus (= C), hypothesizing potential learning effects in single observers over time. **A: No Pathology**; cf. supplementary results for statistical results **B: Pleural Thickening**; cf. supplementary results for statistical results

**
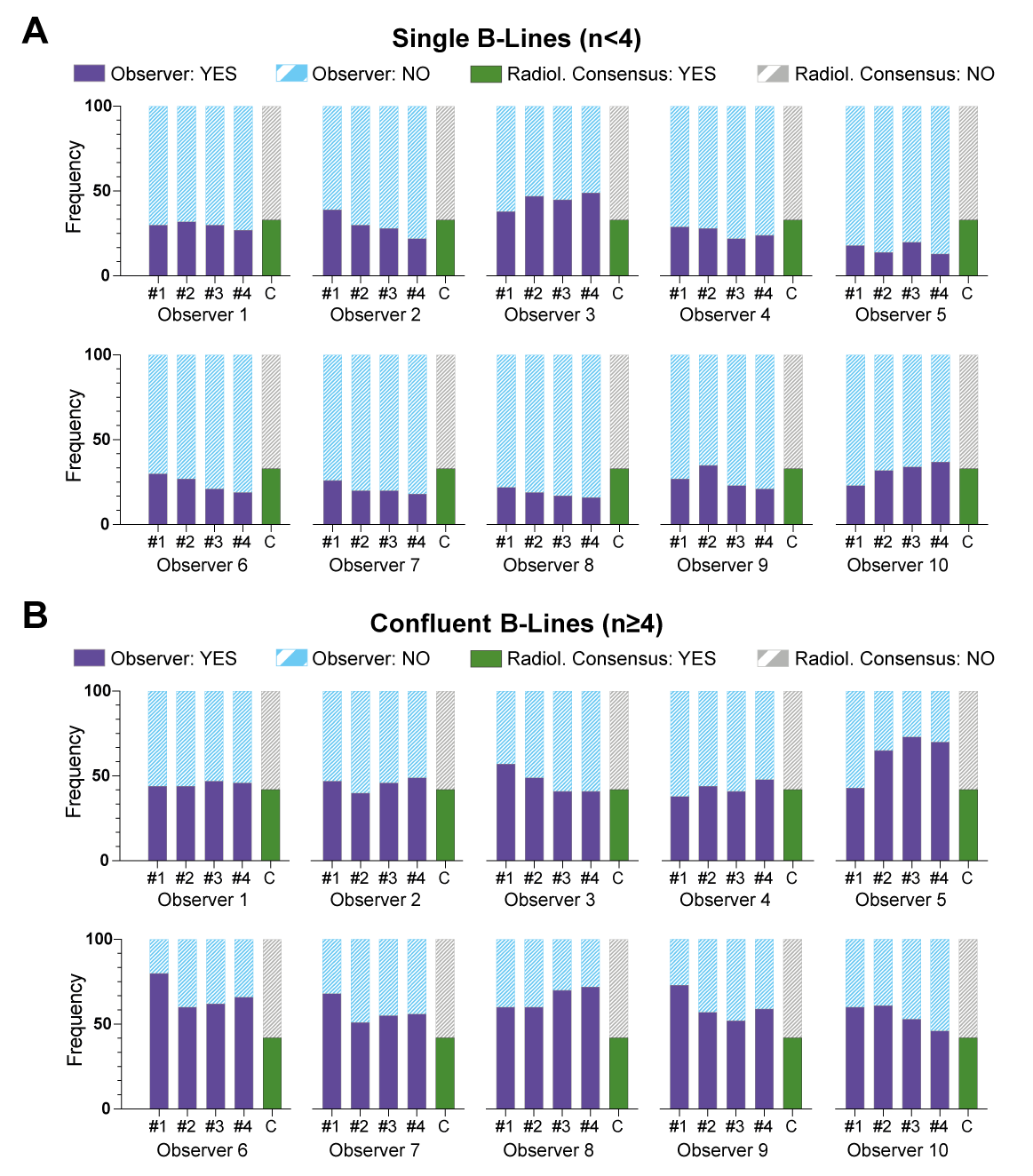
**

**Figure 3-2s**. **Intra-observer learning effect over time (2)**. Graphic representation of count of binary response frequency for specific pathologies in each 100 video loops per instance and observer. We used Cochrane’s-Q-Test for determining intraobserver differences over 4 viewing instances (= #1, #2, #3, #4) and the answer determined by radiological consensus (= C), hypothesizing potential learning effects in single observers over time. **A: Single B-Lines (n<4)**; cf. supplementary results for statistical results **B: Confluent B-Lines (n≥4)**; cf. supplementary results for statistical results

**
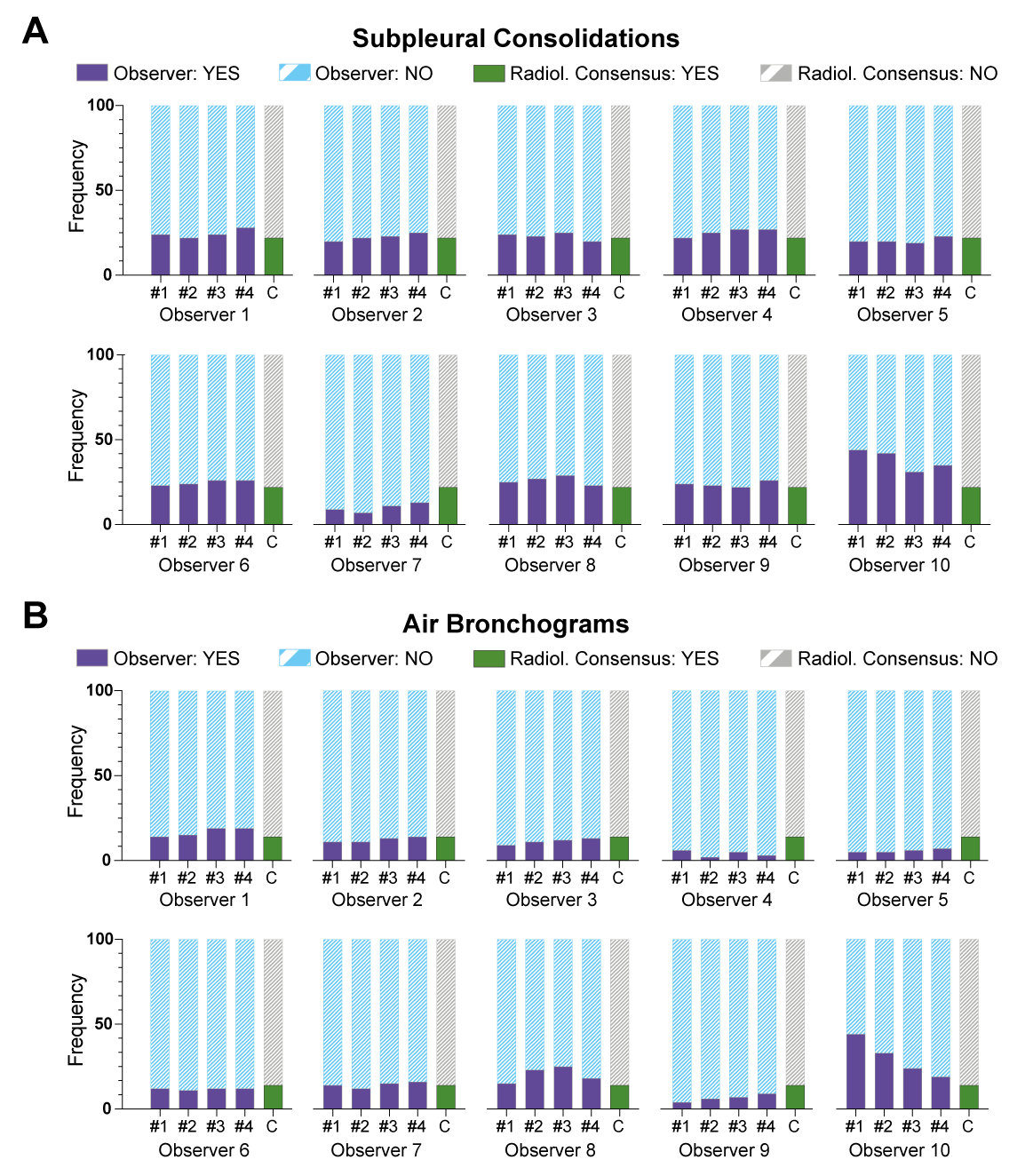
** **Figure 3-3s.** **Intra-observer learning effect over time (3)**. Graphic representation of count of binary response frequency for specific pathologies in each 100 video loops per instance and observer. We used Cochrane’s-Q-Test for determining intraobserver differences over 4 viewing instances (= #1, #2, #3, #4) and the answer determined by radiological consensus (= C), hypothesizing potential learning effects in single observers over time. **A: Subpleural Consolidations**; cf. supplementary results for statistical results **B: Air bronchograms**; cf. supplementary results for statistical results
